# Neural Basis of Video Gaming: A Systematic Review

**DOI:** 10.3389/fnhum.2017.00248

**Published:** 2017-05-22

**Authors:** Marc Palaus, Elena M. Marron, Raquel Viejo-Sobera, Diego Redolar-Ripoll

**Affiliations:** ^1^Cognitive NeuroLab, Faculty of Health Sciences, Universitat Oberta de CatalunyaBarcelona, Spain; ^2^Laboratory for Neuropsychiatry and Neuromodulation, Massachusetts General HospitalBoston, MA, USA

**Keywords:** addiction, cognitive improvement, functional changes, internet gaming disorder, neural correlates, neuroimaging, structural changes, video games

## Abstract

**Background:** Video gaming is an increasingly popular activity in contemporary society, especially among young people, and video games are increasing in popularity not only as a research tool but also as a field of study. Many studies have focused on the neural and behavioral effects of video games, providing a great deal of video game derived brain correlates in recent decades. There is a great amount of information, obtained through a myriad of methods, providing neural correlates of video games.

**Objectives:** We aim to understand the relationship between the use of video games and their neural correlates, taking into account the whole variety of cognitive factors that they encompass.

**Methods:** A systematic review was conducted using standardized search operators that included the presence of video games and neuro-imaging techniques or references to structural or functional brain changes. Separate categories were made for studies featuring Internet Gaming Disorder and studies focused on the violent content of video games.

**Results:** A total of 116 articles were considered for the final selection. One hundred provided functional data and 22 measured structural brain changes. One-third of the studies covered video game addiction, and 14% focused on video game related violence.

**Conclusions:** Despite the innate heterogeneity of the field of study, it has been possible to establish a series of links between the neural and cognitive aspects, particularly regarding attention, cognitive control, visuospatial skills, cognitive workload, and reward processing. However, many aspects could be improved. The lack of standardization in the different aspects of video game related research, such as the participants' characteristics, the features of each video game genre and the diverse study goals could contribute to discrepancies in many related studies.

## Introduction

Nowadays, video gaming is a highly popular and prevalent entertainment option, its use is no longer limited to children and adolescents. Demographic data on video gaming shows that the mean age of video game players (VGPs) (31 years old, as of 2014) has been on the rise in recent decades (Entertainment Software Association, 2014), and it is a common activity among young adults. Moreover, the increasing ubiquity of digital technologies, such as smart-phones and tablet computers, has exposed most of the population to entertainment software in the form of casual video games (VGs) or gamified applications. Therefore, an important segment of society, over 30% in tablet computers and 70% in smart phones, has been exposed to these technologies and can be considered now, in some form, casual gamers (Casual Games Association, 2013).

It is not uncommon to hear both positive and negative health claims related to VGs in the mass media. Most of the time, these are unverified and sensationalist statements, based on “expert” opinions, but lacking evidence behind them. On the other side, as VGs become more complex (due to improvements in computer hardware), they cater to audiences other than children, appealing to older audiences, and VGs have gained prevalence as a mainstream entertainment option. Consequently, the number of people who spend hours daily playing these kinds of games is increasing.

There is interest in knowing the possible effects of long-term exposure to VGs, and whether these effects are generally positive (in the shape of cognitive, emotional, motivation, and social benefits) (e.g., Granic et al., 2014) or negative (exposure to graphic violence, contribution to obesity, addiction, cardio-metabolic deficiencies, etc.) (e.g., Ivarsson et al., 2013; Turel et al., 2016). Moreover, VGs possess a series of intrinsic features which make them suitable for use in experimental procedures: they seem to increase participants' motivation better than tasks traditionally used in neuropsychology (e.g., Lohse et al., 2013) and, in the case of purpose-made VGs, they offer a higher degree of control over the in-game variables.

**Figure 1:**
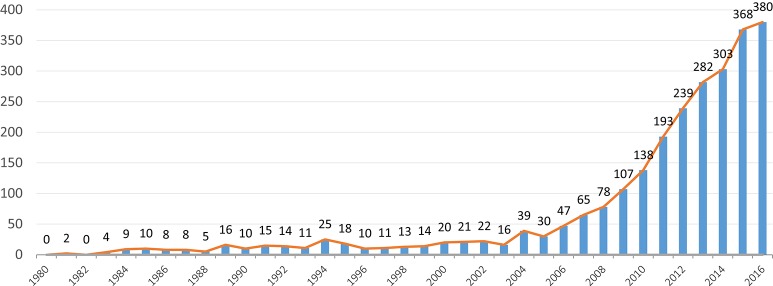
**Increasing trend in VG-related articles**. Since 2005, the average annual growth is around 20%. (Source: MEDLINE).

For all the reasons mentioned above, VGs have recently sparked more scientific interest. The number of publications that study or use some form of gaming has been increasing, since 2005, at a constant rate of 20% per year. While during the 90's around 15 VG-related articles were published per year, in 2015 that number was over 350 (see Figure 1).

However, the concept of VG is extremely heterogeneous and within the category we find a myriad of hardly comparable genres. The behavioral effects and the neural correlates derived from the use of VGs depend both on the nature of the VG, the exposition to the game (hours of game play, age of onset, etc.) (Kühn and Gallinat, 2014), and, to a large extent, the individual characteristics of each participant (Vo et al., 2011).

Furthermore, due to the popularity of VG genres where graphic violence is prevalent (shooters, survival horror, fantasy), many studies have chosen to focus on this variable. Therefore, there is a reasonable amount of scientific literature devoted to the study of violent behaviors and violence desensitization as a consequence of violence in VGs (e.g., Wang et al., 2009; Engelhardt et al., 2011). Lastly, in particular since the emergence of online VG play, there are concerns about the addictive properties of VGs, akin to gambling and substance abuse, consequently making it another recurrent topic in the literature (e.g., Young, 1998).

For the time being, this whole body of knowledge is a complex combination of techniques, goals and results. On one hand, there are articles which study the effects of VG exposure over the nervous system and over cognition (e.g., Green and Seitz, 2015); it seems that there is solid evidence that exposure to certain kinds of VGs can have an influence on behavioral aspects, and therefore, we should be able to appreciate changes in the neural bases (Bavelier et al., 2012a). Actually, assessing the cognitive and behavioral implications of VG exposure has already been the object of study in recent systematic reviews and meta-analysis that used neuropsychological tasks to measure the influence of these games in healthy individuals. This is highly relevant since they evaluate the possible transfer effects of VG training to wider cognitive domains, providing a global perspective on how experimental and quasi-experimental designs differ in the size of the effect depending on the cognitive function (Powers et al., 2013), and how aging interferes with cognitive training by means of computerized tasks (Lampit et al., 2014) and VGs (Toril et al., 2014; Wang et al., 2016). Knowledge obtained about transfer effects is very important since it allows us to establish a link between VGs and cognition, indirectly helping us understand its neural basis, which in this case acts as a bridge between them. From an applied perspective, this knowledge can be used to design more effective rehabilitation programs, especially those focusing on older populations, keeping the most useful components and reducing those which are shown to have less benefits.

On the other hand, VGs have been used as a research tool to study the nervous system. In this group of studies, it is common to find exposure to VGs as the independent variable, especially in most studies that use unmodified commercial VGs. However, it is not unusual to employ custom designed VGs, such as the widely used Space Fortress, where in-game variables can be fine-tuned to elicit certain mental processes in consonance with the research hypothesis (e.g., Smith et al., 1999; Anderson et al., 2011; Prakash et al., 2012; Anderson et al., 2015). Nevertheless, in both cases, the study of the VG exposure over the nervous system and the use of VGs as a research tool, VGs are used to obtain information about the underlying neural processes relevant to our research interest.

As yet there is no systematic review on this topic. The aim of this article is to gather all the scientific information referring to neural correlates of VGs and synthesize the most important findings. All articles mentioning functional and structural changes in the brain due to video gaming will be analyzed and information about the most relevant brain regions for each kind of study will be extracted; the main objective of many VG-related articles is not to study their neural correlates directly. Studies focusing on the addictive consequences or the effects of violence will be categorized independently.

Our final goal is to highlight the neural correlates of video gaming by making a comprehensive compilation and reviewing all relevant scientific publications that make reference to the underlying neural substrate related to VG play. This is the first effort in this direction that integrates data regarding VGs, neural correlates and cognitive functions that is not limited to action-VGs or cognitive training programs, the most frequently found research topics.

## Methods

In order to structure reliably the gathered information in this systematic review, the guidelines and recommendations contained in the PRISMA statement (Liberati et al., 2009) have been followed.

### Eligibility criteria

All articles which included neural correlates (both functional and structural) and included VG play in the research protocol or studied the effects of exposure to VGs were included in the review. Both experimental and correlational studies were included. No restrictions regarding publication date were applied.

Healthy participants of any age and gender were considered. Studies include both naive and experienced VG participants. Participants that reported gaming addiction or met criteria for internet gaming disorder (IGD) were also included in the review owing to the interest in observing neural correlates in these extreme cases. Other pathologies were excluded in order to avoid confounding variables.

Articles employing several methodologies were included. These can be organized into three main groups: studies where naive participants were trained in the use of a VG against a control group, studies comparing experienced players vs. non-gamers or low-experience players, and studies comparing differential characteristics of two VG or two VG genres.

The primary outcome measures were any kind of structural and functional data obtained using neuroimaging techniques including computerized tomography (CT) scan, structural magnetic resonance imaging (MRI), functional MRI (fMRI), positron emission tomography (PET), single-photon emission computed tomography (SPECT), magneto encephalography (MEG), transcranial direct current stimulation (tDCS), electroencephalogram (EEG), event-related potentials (ERP), event-related spectral perturbation (ERSP), steady state visually evoked potential (SSVEP), Doppler, and near-infrared spectroscopy (NIRS), following or related to VG use.

### Information sources

Academic articles were located using two electronic databases: MEDLINE and Web of Science, and by scanning reference lists in other studies in the same field. Only the results from these two databases are reported since results from other sources (Scopus, Google Scholar) did not provide any relevant new results. The search was not limited by year of publication and only articles published in English, Spanish, or French were considered for inclusion. The first studies relevant to the topic are from 1992, while the most recent studies included in this review were published in February 2016.

### Search

A systematic search was performed using a series of keywords which were expected to appear in the title or abstract of any study containing neural correlates of VGs. These keywords were grouped in two main categories. First of all, a group of keywords trying to identify articles which used VG as a technique or as a study goal. These keywords included search terms related to “video games” proper (in different orthographic variants), types of players (casual, core, and hardcore gamers) and references to serious gaming. In second place, two groups of keywords were used to detect articles which studied the neural basis: (1) keywords related to anatomical features, such as structural or functional changes, gray, or white matter (WM) volumes, cortical features, and connectivity and (2) keywords which mentioned the neuroimaging technique used to obtain that data, such as EEG, MRI, PET, or NIRS. (See Appendix)

### Study selection

**Figure 2:**
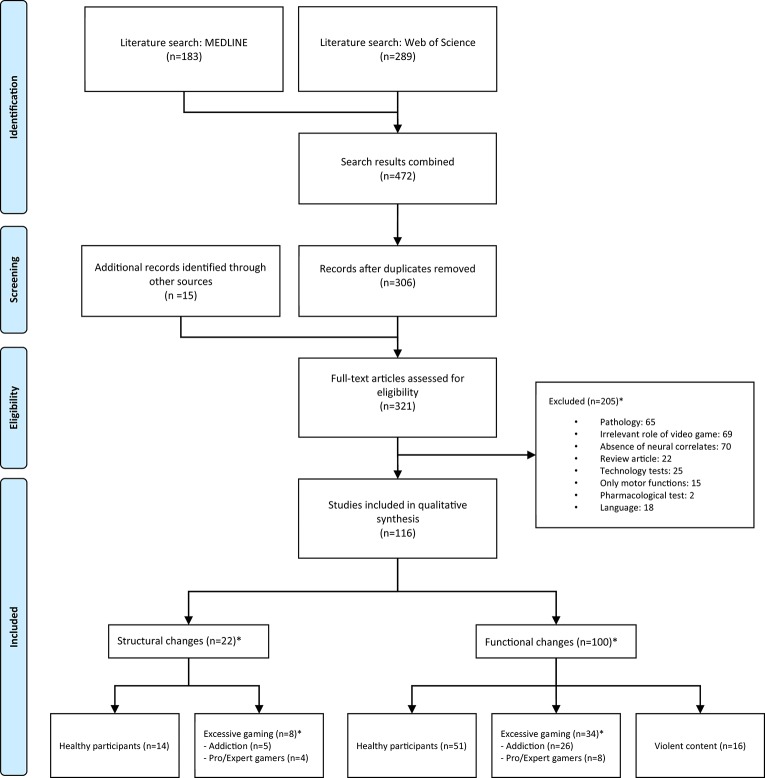
**Study selection diagram flow**. ^*^Articles in these sections may not be mutually exclusive.

Due to the large amount of results obtained by the previous search terms, strict exclusion criteria were applied to limit the final selection of studies. The same criteria were applied in a standardized way by two independent reviewers, and disagreements between reviewers were resolved by consensus. Due to high variability in the terminology and the diversity of keywords used in the search, a large number of false positive studies (65% of items found) appeared during the review process (see Figure 2).

By performing a search using standardized terms, a list of studies from the two databases was extracted. A large number of studies (62% of those that met the inclusion criteria) were found to be duplicates in both databases, so a careful comparison was made in order to merge the references.

No unpublished relevant studies were considered. Studies relevant to the topic but not published in peer-reviewed journals, such as conference posters and abstracts were considered.

### Data collection process

All the relevant information was classified in a spreadsheet, according to the variables listed below. Variables related to violence and abuse of VGs were also categorized, since a significant portion of the studies focused on these behaviors. A small number of articles (*n* = 7) were found in sources other than the two databases, mainly through references in other articles.

For each study, the following data was extracted: (1) characteristics of the sample, including sample size, average age and range, inclusion and exclusion criteria, and gaming experience; (2) aim of the study, specially noting if it is focused on gaming abuse or exposure to violent content; (3) name and genre of the VG used during the study, if applicable; (4) study design; (5) main neuroimaging technique applied in the study, and whether the technique was applied while participants played; (6) functional and structural neural correlates observed in the study. Studies were then classified in several groups as to whether they provided structural or functional data, and whether they addressed violent or addictive aspects.

Moreover, in order to understand the outcomes derived from the neural correlates, most of the studies establish a connection between these correlates and their cognitive correspondence, either by directly measuring the outcomes using cognitive tasks and questionnaires, or by interpreting their results based on existing literature.

In the discussion section of this review, we attempted to summarize the main findings by associating the neural changes to their cognitive and behavioral correspondences. Whereas, in many cases the original articles provided their own explanation for the phenomena, we also worked on integrating the general trends from a cognitive perspective. We therefore indicate which studies provide and interpret empirical cognitive or/and behavioral data (non-marked), those which discuss cognitive or/and behavioral implications without assessing them (marked with ^*^), and those which did not provide any cognitive nor behavioral information (marked with ^**^).

## Results

### Study selection

The combined search of MEDLINE and Web of Science provided a total of 306 unique citations. Of these, 205 studies were discarded because they did not seem to meet the inclusion criteria after reviewing the abstract. The main reasons for exclusion were: being a review article (*n* = 22), absence of neural correlates (*n* = 70), presence of pathology in the participants (*n* = 65), not being related to VGs or using simple computerized tasks which could not be considered VGs (*n* = 69), testing of new technologies in which the brain correlates were a mere by-product (*n* = 25), articles focused on motor functions (*n* = 15), pharmacological studies (*n* = 2), and finally, articles in languages other than English, Spanish, or French (*n* = 18). Excluded articles often met more than one exclusion criteria. As mentioned in the eligibility criteria, an exception were those articles in which the pathology consisted of gaming overuse or addiction and articles which featured psychopathology and included groups of healthy participants from whom neural data was provided.

Fifteen extra articles that met the inclusion criteria were found after examining the contents and following the references in the previously selected studies. As expected, articles written in English comprised the vast majority; among the rest (8.9%), 10 of them (4.9%) were discarded from the review solely for language reasons. No unpublished relevant studies were considered. Studies relevant to the topic but not published in peer-reviewed journals, such as conference posters and abstracts were considered. Ultimately, a total of 116 studies were identified for inclusion in the review (see flow diagram in Figure 2).

Most studies (*n* = 100; 86.2%) provided functional data, while only 22 (18.9%) of them studied structural changes in the brain. A few (*n* = 6; 5.2%) provided both structural and functional data. A significant number of the studies focused their attention on excessive playing or VG addiction. That was the case for 39 (33.6%) of the reviewed articles, so we considered it appropriate to analyze them in their own category. Likewise, 16 studies (13.8%) focusing on the violent component of VGs were also placed in their own category. These categories were not always exclusive, but there was only one case where the two criteria were met. (See Table 1 for a breakdown by category).

**Table 1:** **Article breakdown by category**.

	**Structural**	**Functional**	**Both**	**Total**
All	22	100	6	116
Healthy (Non excessive, non-violent)	14	51	3	62
Excessive gaming	8	34	3	39
Excessive gaming, IGD	5	26	2	29
Excessive gaming, Non-IGD	3	8	1	10
Violence	0	16	0	16
Violence + Excessive gaming	0	1	0	1
	**Only structural**	**Only functional**		
	16	94		

*IGD, Internet Gaming Disorder*.

### Characteristics of included studies

Based on their methodology, studies in this review could be classified as experimental (*n* = 54; 46.6%), randomly assigning the participant sample to the experimental groups, and quasi-experimental (*n* = 62; 53.4%), where the groups were usually constructed according to the participants' characteristics. While studies involving excessive gaming almost always followed a quasi-experimental design comparing experienced gamers against low-experience VG players, articles studying normal gaming and the effects of violence exposure used both experimental and quasi-experimental designs. A fraction of the studies (*n* = 15; 13%), both experimental and quasi-experimental, compared the results to a baseline using a pretest-posttest design. That was the case for most studies involving a training period with VGs.

The cumulative sample included in this review exceeds 3,880 participants. The exact number cannot be known since participants could have been reused for further experiments and in some cases the sample size was not available. Most studies used adolescents or young adults as the primary experimental group, since that is the main demographic target for video gaming. In many cases, only male participants were accepted. In the cases where VG experience was compared, the criteria varied greatly. For the low video gaming groups, VG usage ranged from <5 h/week to none at all. For the usual to excessive VG groups, it could typically start at 10 h/week. In some cases, where the level of addiction mattered, the score in an addiction scale was used instead.

In more than half of the studies (*n* = 67; 57.8%) participants actually played a VG as part of the experimental procedure. In the rest, either neural correlates were measured in a resting-state condition or VG related cues were presented to the participants during the image acquisition.

Structural changes in the gray matter (GM) were measured in the form of volumetric changes, whereas WM was assessed using tractography techniques. Functional changes were typically measured comparing activation rates for different brain regions. Nearly half (*n* = 55; 47.4%) of the assessed studies used fMRI as the neuroimaging technique of choice, while other functional techniques remained in a distant second place. Functional connectivity was assessed in several studies employing resting-state measures. EEG in its multiple forms was also widely used (*n* = 32; 27.6%) to obtain functional data, either to measure activation differences across regions or in the form of event related potentials. (See Table 2 for a breakdown by neuroimaging technique).

**Table 2:** **Neuroimaging techniques used in the reviewed studies**.

**Technique**	**N**	**%**
Electrophysiological methods	32	27.6
EEG (standard)	13	11.2
ERP	16	13.8
ERSP	1	0.9
SSVEP	2	1.7
MRI	70	60.3
MRI (structural)	15	12.9
fMRI	55	47.4
NIRS	8	6.9
SPECT	2	1.7
PET	2	1.7
Doppler	1	0.9

*EEG, Electroencephalography; ERP, Event-related potentials; ERSP, Event-related spectral Dynamics; fMRI, Functional magnetic resonance imaging; MRI, Magnetic resonance imaging; NIRS, Near-infrared spectroscopy; PET, Positon emission tomography; SPECT, Single-photon emission computed tomography; SSVEP, Steady-state visual evoked potential*.

The high variability in the study designs, participants and objectives meant we focused on describing the studies, their results, their applicability, and their limitations on a qualitative synthesis rather than meta-analysis.

### Structural data

Data regarding structural changes following VG use was available from 22 studies, fourteen of which provided structural data for more than 800 participants that had a normal VG use and included both VGPs and non-VGPs (see Table 3). The remaining eight studies examined aspects concerning the excessive or professional use of VG (see Table 4).

**Table 3:** **Studies providing structural data dealing with healthy, non-expert participants**.

**Ref**.	**Year**	***N***	**Age**	**Sample**	**VP experience**	**VG genre**	**Technique**	**Design**	**Neural correlates**
Erickson et al., 2010	2010	39	(18–28)	Healthy young adults	<3 h/week	Action, shooter	MRI	Experimental (randomized)	Predictors of skill acquisition: ▴ DS ▴ VS
Basak et al., 2011	2011	20	70.1 ± 4.81	Healthy older adults	Low VGP (<1 h/week)	Real time strategy	MRI	Quasi-experimental (one group pretest-posttest)	Predictors of skill acquisition: ▴ MFG (left) ▴ PoCG (left) ▴ dlPFC (left) ▴ ACC (right) ▴ Cerebellum
Vo et al., 2011	2011	34	(18–28)	Healthy young adults	Low or Non-VGP	Action, shooter	MRI	Experimental (crossover)	Skill acquisition: ▴ DS
Colom et al., 2012	2012	20	18.95 ± 2.65	Healthy young adults, female	Low or Non-VGP	Puzzle, Brain training	MRI (DTI)	Experimental (randomized with pretest)	After VG training: Gray matter: ▴ PFC (BA9 & BA10) ▴ Small temporal and parietal regions White matter: ▴ HC cingulum ▴ ILF
Kühn et al., 2013	2013	48	24.1 ± 3.8	Healthy young adults	Low or Non-VGP	Action, 3D platforms	MRI	Experimental (randomized with pretest)	After VG training: ▴ HC (right) ▴ dlPFC (right) ▴ Cerebellum
McGarry et al., 2013	2013	7	(60–85)	Healthy older adults	–	Real time strategy	MRI	Quasi-experimental (one group pretest-posttest)	After VG training: ▴ SPG ▴ Lateral IFG ▴ PrCG ▴ FG
Kühn and Gallinat, 2014^*^	2014	62	28.4 ± 6.07	Healthy young adults, male	Low or Non-VGP (*n* = 48) Excessive (*n* = 9) IGD (*n* = 5)	–	MRI	Quasi-experimental (retrospective)	VG experience: ▴ Entorhinal cortex ▴ HC Game genre: ▴ Entorhinal cortex
Kühn et al., 2014^*^	2014	152	14.4 ± 0.03	Healthy adolescents	12.6 ± 12.9 h/week	–	MRI	Quasi-experimental (retrospective)	VG experience: ▴ dlPFC (left) ▴ MFG(left) ▴ SFG (left) ▴ FEF (left)
Strenziok et al., 2013	2014	–	(>60)	Healthy older adults	–	Real time strategy Puzzle, Brain training	MRI (DTI)	Experimental (randomized)	After VG training: ▾ Splenium of CC
Strenziok et al., 2014	2014	42	69.21 ± 4.9	Healthy older adults	–	Action, shooter Real time strategy Puzzle, Brain training	MRI (DTI)	Experimental (randomized with pretest)	After VG training (white matter AD): All 3 groups: ▴ Lingual gyrus (left) ▴ Thalamus (right) Brain training vs. Action shooter: ▴ TO junction (right) Brain training vs. Strategy: ▴ POT junction (right)
Szabó et al., 2014^**^	2014	56	36.8 ± 10.3	Healthy adults	Low or Non-VGP	Action, 3D platforms	MRI	Experimental (randomized with pretest)	After VG training: ▴ HC (right)
Zhang et al., 2015	2015	45	16.9 ± 2.2 (VGP) 17.1 ± 1.3 (Non-VGP)	Healthy adolescents, male	VGP (19 h/week) Low or Non-VGP (<2 h/week)	Racing Role playing, MMORPG Dance Action, First Person Shooter	MRI (DTI)	Quasi-experimental (retrospective)	VGP vs. Non-VGP (White matter FA): ▴ CST (left) ▴ SLF (left) ▴ ILF ▴ IFOF
Kim Y. H. et al., 2015^*^	2015	31	29.0 ± 4.1	Healthy young adults	VGP (>3 h/week) Non-VGP (<10 h/year)	Real time strategy	MRI (DTI)	Quasi-experimental (with control group)	VGP vs. Non-VGP: White matter connectivity: ▴ EC (right) & Visual cortex ▴ IFG (right) & ACC
Takeuchi et al., 2016	2016	240	11.1 ± 2.7	Healthy children	0.8 ± 0.75 h/week	–	MRI (DTI)	Quasi-experimental (cross-sectional)	VG experience: ▴ PFC (bilateral) (GM & WM) ▴ ACC ▴ Lateral & Medial temporal cortex ▴ BG ▴ FG ▾ Genu of de CC (Specific areas) ▾ Body of the CC ▾ ACR (bilateral) ▾ SCR (right)
Takeuchi et al., 2016	2016	189	(5.7–16.6)	Healthy children	–	–	MRI (DTI)	Quasi-experimental (longitudinal)	VG experience: ▴ Cluster 1: BG (Gm & WM) (left) Medial temporal lobe (left) Thalamus (bilateral) ▴ Cluster 2: Insula (right) Putamen (right) Thalamus (right) ▴ Cluster 3: MTG & ITC (left) FG Occipital lobe (left)

ACC, Anterior cingulate cortex; ACR, Anterior corona radiata; AD, Axial diffusivity; BA, Brodmann area; BG, Basal ganglia; CC, Corpus callosum; CST, Corticospinal tract; dlPFC, Dorsolateral prefrontal cortex; DS, Dorsal striatum; DTI, Diffusion tensor imaging; EC, External capsule; FA, Fractional anisotropy; FEF, Frontal eye fields; FG, Fusiform gyrus; GM, Gray matter; HC, Hippocampus; IFG, Inferior frontal gyrus; IFOF, Inferior frontooccipital fasciculus; IGD, Internet Gaming Disorder; ILF, Inferior longitudinal fasciculus; ITC, Inferior temporal cortex; MFG, Middle frontal gyrus; MRI, Magnetic resonance imaging; MTG, Middle temporal gyrus; PFC, Prefrontal cortex; PoCG, Post central gyrus; POT, Parieto-occipito-temporal; PrCG, Pre-central gyrus; SCR, Superior corona radiata; SFG, Superior frontal gyrus; SLF, Superior longitudinal fasciculus; SPG, Superior parietal gyrus; TO, Temporo-occipital; VG, Video game; VGP, Video game player; VS, Ventral striatum; WM, White matter. Articles marked with an asterisk

(*) discuss cognitive implications without directly assessing this dimension. Articles marked with a double asterisk

(**) *did not provide either empirical cognitive data nor discuss cognitive implications. The rest of the articles (non-marked) have measured cognitive correlates with specific tasks*.

**Table 4:** **Studies providing structural data dealing with VG experts or excessive gaming**.

**Ref**.	**Year**	***N***	**Age**	**Sample**	**VG experience**	**VG genre**	**Technique**	**Design**	**Neural correlates**
Han et al., 2012a	2012	55	20.9 ± 2.0 (IGD) 20.8 ± 1.5 (Pro) 20.9 ± 2.1 (Control)	Young adults, male	IGD (9.0 ± 3.7 h/day) Professional VGP (9.4 ± 1.6 h/day) Low or Non-VGP (1.0 ± 0.7 h/day)	Real time strategy	MRI	Quasi-experimental (with control group)	IGD vs. Control: ▴ Thalamus GM (left) ▾ ITG (bilateral) ▾ MOG (right) ▾ IOG (left) Professional vs. Control: ▴ CG (left) ▾ MOG (left) ▾ ITG (right) Professional vs. IGD: ▴ CG (left) ▾ Thalamus (left)
Hou et al., 2012^*^	2012	14	20.40 ± 2.30 (IGD) 20.44 ± 1.13 (Control)	Young adults	IGD (>8 h/day) Low or Non-VGP (<5 h/day)	–	SPECT	Quasi-experimental (with control group)	IGD vs. Control: ▾ Striatum volume ▾ Striatum weight ▾ Striatum/whole brain ratio
Hyun et al., 2013	2013	23	19.8 ± 1.7	Healthy young adults, male	Professional VGP (9.2 ± 1.6 h/day)	Real time strategy	MRI	Quasi-experimental	Career length: ▴ SFG (right) ▴ SPG (right) ▴ PrCG (right) Winning rates: ▴ PFC
Tanaka et al., 2013	2013	50	24.1 ± 2.9 (Experts) 22.4 ± 3.42 (Control)	Healthy young adults, male	Expert VGP (21.4 ± 10.0 h/week) Low or Non-VGP (<2 h/week)	Action, fighting	MRI	Quasi-experimental (with control group)	Experts vs. Non-experts: ▴ PPC (right)
Yuan et al., 2013a	2013	36	19.4 ± 3.1 (IGD) 19.5 ± 2.8 (Control)	Young adults, male	IGD (10.2 ± 2.6 h/day) Low or Non-VGP (0.8 ± 0.4 h/day)	Role playing, MMORPG	MRI	Quasi-experimental (with control group)	IGD vs. Control: ▴ Precentral cortex (left) ▴ PCu ▴ MFG ▴ ITC ▴ MTG ▾ lOFC (left) (Impaired task performance) ▾ Insula ▾ Lingual gyrus ▾ PoCG (right) ▾ Entorhinal cortex ▾ IPC
									IGD duration: ▴ Precentral cortex (left) ▴ PCu ▴ Lingual gyrus
Xing et al., 2014	2014	34	19.1 ± 0.7 (IGD) 19.8 ± 1.3 (Control)	Young adults	IGD (9.5 ± 1.3 h/day; 65.7 ± 11.6 IAT) Control (2.2 ± 1.4 h/day; 29.2 ± 4.5 IAT)	Action, Real time strategy	MRI (DTI)	Quasi-experimental (with control group)	IGD vs. Control (FA): ▾ SN tract (right)
Gong et al., 2015^*^	2015	57	23.26 ± 0.4 (Experts) 22.36 ± 0.38 (Amateurs)	Healthy young adults	Expert VGP (46.67 ± 2.1 h/week) Amateur VGP (14.2 ± 1.1 h/week)	Action, Real time strategy	MRI	Quasi-experimental (with control group)	Experts vs. Amateurs): ▴ Insula (left) ▴ Short insular gyri ▴ Long insular gyrus ▴ Central sulcus
Jin et al., 2016^*^	2016	46	19.12 ± 1.05 (IGD) 18.76 ± 1.81 (Control)	Adolescents	IGD (5.32 ± 2.10 h/day) Low or Non-VGP (2.07 ± 1.39 h/day)	Action, Real time strategy	MRI	Quasi-experimental (with control group)	IGD vs. Control: ▾ dlPFC ▾ OFC ▾ ACC ▾ SMA (right)

ACC, Anterior cingulate cortex; CG, Cingulate gyrus; dlPFC, Dorsolateral prefrontal cortex; DTI, Diffusion tensor imaging; FA, Fractional anisotropy; GM, Gray matter; IGD, Internet gaming disorder; IOG, Inferior occipital gyrus; IPC, Inferior parietal cortex; ITC, Inferior temporal cortex; lOFC, Lateral orbitofrontal cortex; MFG, Middle frontal gyrus; MMORPG, Massively multiplayer online role-playing game; MOG, Middle occipital gyrus; MRI, Magnetic resonance imaging; MTG, Middle temporal cortex; OFC, Orbitofrontal cortex; PCu, Precuneus; PFC, Prefrontal cortex; PoCG, Post-central gyrus; PPC, Posterior parietal cortex; PrCG, Pre-central gyrus; SFG, Superior frontal gyrus; SMA, Supplementary motor area; SN, Salience network; SPECT, Single-photon emission computed tomography; SPG, Superior parietal gyrus; VGP, Video game player. Articles marked with an asterisk

(*) discuss cognitive implications without directly assessing this dimension. Articles marked with a double asterisk

(**) did not provide either empirical cognitive data nor discuss cognitive implications. The rest of the articles (non-marked) have measured cognitive correlates with specific tasks.

In studies dealing with healthy, non-addicted participants, eight studies used MRI to provide structural information for the GM, while six focused on the WM using diffusion tension imaging (DTI).

Three studies compared lifetime VG experience prior to the study, while the rest used a training paradigm where participants were exposed to a VG during the experimental sessions prior to the neuroimaging procedure and compared to a baseline. Seven studies provided WM integrity data using the DTI technique while the rest analyzed cortical thickness variations using regular structural MRI.

The most researched areas in studies examining volumetric differences found relevant changes in prefrontal regions, mainly the dorsolateral prefrontal cortex (dlPFC) and surrounding areas, superior and posterior parietal regions, the anterior cingulate cortex (ACC), the cerebellum, the insula, and subcortical nuclei, as well as the striatum and the hippocampus. In addition to this, structural connectivity studies observed changes in virtually all parts of the brain, such as in fibers connecting to the visual, temporal and prefrontal cortices, the corpus callosum, the hippocampus, the thalamus, association fibers like the external capsule, and fibers connecting the basal ganglia.

### Functional data

A 100 articles provided functional data combined with VG use. Of these, around half (*n* = 51) were studies which did not include violence or addiction elements (See Table 5). A third (*n* = 34) corresponded to articles aiming at understanding the neural bases of IGD (See Table 6), often drawing parallels with other behavioral addictions and trying to find biomarkers for VG addiction. The rest (*n* = 16) were devoted to study the effects of violence exposure in VGs (See Table 7). In total, these studies provided functional data for 3,229 experimental subjects, including control groups. Note that there is some overlap with the structural section, since a few (*n* = 6) studies provided both structural and functional data.

**Table 5:** **Studies providing functional data dealing with healthy, non-expert participants, without violent content**.

**Ref**.	**Year**	***N***	**Age**	**Sample**	**VG experience**	**VG genre**	**Technique**	**Design**	**Neural correlates**
Kelley et al., 1992^**^	1992	21	31.4 ± 7.8	Healthy adults	–	Breakout	Doppler	Experimental (crossover)	VG play vs. Baseline: ▴ MCA (bilateral) ▴ PCA (left)
Brookings et al., 1996	1996	8	(21–29)	Healthy adults	Air traffic controllers	Simulation	EEG	Experimental (crossover)	Task difficulty (measured in Theta power): High vs. Low difficulty: ▴ F8, C3, Cz, T4, P3, Pz, P4 Medium vs. Low difficulty: ▴ C3, P3, Pz Overload condition vs. Low difficulty: ▴ F3, C3, Pz Overload condition vs. Medium difficulty: ▴ T6, O2 Overload condition vs. High difficulty: ▴ F7, F3, Fz, C3
Koepp et al., 1998^**^	1998	8	(36–46)	Healthy adults, male	–	Action	PET	Quasi-experimental (with pretest)	VGP vs. baseline: ▾ Striatum (dopamine binding) Performance level: ▾ VS (dopamine binding)
Pellouchoud et al., 1999^*^	1999	7	(9–15)	Healthy children	–	Puzzle	EEG	Experimental (crossover)	Gameplay vs. resting: ▴ Frontal midline theta (6–7 Hz) ▾ Posterior alpha (9–12 Hz) ▾ Central mu (10–13 Hz)
Smith et al., 1999	1999	6	(22–25)	Healthy young adults	–	Action, shooter	EEG	Quasi-experimental (with pretest)	VG Post vs. Pre: ▾ Central alpha waves ▴ Primary motor cortex alpha waves ▴ Frontal midline theta waves
Izzetoglu et al., 2004^*^	2004	8	(18–50)	Healthy adults	–	Action, strategy	NIRS	Experimental (crossover)	VG difficulty: ▴ dlPFC (bilateral)
Matsuda and Hiraki, 2004^*^	2004	6	(23–29)	Healthy young adults	–	Action, First Person Shooter Rhythm Puzzle	NIRS	Experimental (crossover)	VG play vs. rest: ▾ dPFC Viewing VG or non-VG images: ▾ dPFC Fast vs. slow finger tapping: ▴ dPFC Left vs. right finger tapping: ▴ dPFC
Matsuda and Hiraki, 2006^*^	2006	13	(7–14)	Healthy children	–	Action, fighting Puzzle	NIRS	Experimental (crossover)	Children (VG play vs. rest): ▾ dPFC Children vs. Adults (VG play): = dPFC
Nagamitsu et al., 2006^**^	2006	12	8 (7–10) (children) 34 (26–44) (adults)	Healthy children Healthy adults	Low and High VGP (>2 h/day) Non-VGP	Action, 2D Platforms	NIRS	Quasi-experimental (with control group)	During VG play: ▴ PFC (bilateral) in 4 adults ▾ PFC (bilateral) in 2 children ▴ PFC & Motor cortex (bilateral) correlation
Salminen and Ravaja, 2007^**^	2007	25	23.8	Healthy young adults	VGP (>once a month)	Action, 3D Platforms	EEG	Experimental (crossover)	While playing: Picking up item: ▾ Central theta waves ▾ Frontal high alpha waves ▴ Frontal beta waves Falling: ▾ Central theta ▴ Fronto-central beta waves Reaching goal: ▴ Parietal theta waves ▾ Frontal low alpha waves ▴ Frontal high alpha waves ▾ Central high alpha waves ▴ Parietal high alpha waves ▾ Frontal beta waves ▴ Central beta waves
Sheikholeslami et al., 2007^**^	2007	2	–	Healthy participants	–	Sports	EEG	Quasi-experimental (with pretest)	Gaming vs. resting: ▴ Frontal midline theta waves ▾ Parietal alpha waves & slow increase
Corradi-Dell'Acqua et al., 2008^*^	2008	17	–	Healthy young adults	–	Custom VG	fMRI	Experimental (factorial design)	VG character controlled synchronously: Agency vs. Control: ▴ MCG (left) ▴ MFG Agency vs. Control (when changing spatial positions): ▴ POT junction (right)
Russoniello et al., 2009	2009	69	–	–	–	Puzzle	EEG	Experimental (randomized)	VGP vs. Control: ▾ Frontal alpha waves (left)
Bailey et al., 2010	2010	51	(18–33)	Healthy young adults	Low VGP (1.76 ± 4.75 h/week) High VGP (43.4 ± 16.0 h/week)	Action	EEG (ERP)	Quasi-experimental (with control group)	High VGP vs. Low VGP: ▾ Medial frontal negativity amplitude ▾ Frontal slow wave amplitude
Han et al., 2010b^*^	2010	21	24.1 ± 2.6	Healthy young adults	Low VGP (<1 h/day) High VGP (>1 h/day)	Action, fighting	fMRI	Quasi-experimental (with control group and pretest)	Excessive VGP vs. Control: ▴ ACC ▴ OFC
Anderson et al., 2011^*^	2011	20	23.6	Healthy young adults	VGP (low, medium and high)	Action, shooter	fMRI	Experimental (crossover)	During VG play: ▴ Hand motor regions (bilateral) ▴ ACC ▴ PPC ▴ LIPFC ▴ CN ▴ FG
Maclin et al., 2011	2011	39	(19–29)	Healthy young adults	Low or Non-VGP (<3 h/week)	Action, shooter	EEG (ERP)	Experimental (crossover)	Post vs. Pre-training ▾ P300 amplitude (VG “hits”) ▾ P300 amplitude (oddball tones) ▴ P300 amplitude (VG “enemies”) ▾ Delta power (VG “hits”) ▴ Delta power (oddball tones) ▴ Alpha power (VG “hits”)
									▴ Alpha power (oddball tones) ▴ Delta power (VG “enemies”) ▴ Alpha power (VG “enemies”) ▴ Parietal (Pz) theta power (VG “enemies”) ▴ Parietal (Pz) delta power (VG “enemies”) Oddball task inside VG vs outside game ▾ P300 amplitude
Mishra et al., 2011	2011	41	21 (VGP) 24 (Non-VGP)	Healthy young adults, male	VGP (9.0 ± 2.7 h/week) Non-VGP (0 h/week)	Action	EEG (SSVEP)	Quasi-experimental (with control group)	VGP vs. Non-VGP: ▴ Suppression SSVEP to unattended peripheral sequences ▴ P300 amplitude
Bavelier et al., 2012a	2012	26	20.50	Healthy young adults	VGP (>5 h/week) Low or Non-VGP (<5 h/week)	Action, First Person Shooter	fMRI	Quasi-experimental (crossover, with control group)	As attentional demands increased: Non-VGP vs. VGP: ▴ FPN
Cole et al., 2012^*^	2012	57	25.3 ± 9.4	Healthy young adults	–	Action, shooter	fMRI	Experimental (randomized)	VG onset: ▴ CN ▴ NAcc ▴ PHG VG gameplay: ▴ Thalamus ▴ Posterior insula ▴ Putamen ▴ Motor regions ▾ Parietal cortex ▾ Medial PFC VG offset gameplay: ▴ Anterior insula ▴ ACC VG group vs. Control: ▴ CN ▴ NAcc ▴ PHG
Lee H. et al., 2012	2012	75	21.57 ± 2.58	Healthy young adults	Low or Non-VGP (<4 h/week)	Action, shooter	fMRI	Experimental (randomized)	Full emphasis vs. Hybrid variable-priority training (post-training): ▴ PCu (left) ▴ Lateral occipital cortex (left) ▴ Intracalcarine cortex (left) ▴ SFG (right) Post vs. Pre training: ▾ Intracalcarine cortex (bilateral) ▾ Lingual gyrus (bilateral)
									▾ Lateral occipital cortex (bilateral) Hybrid variable-priority training vs Control: ▾ dlPFC VG skill improvement: ▾ Intracalcarine cortex (right)
Han et al., 2012a^*^	2012	19	20.5 ± 1.5	Healthy young adults, male	–	Action, First Person Shooter	fMRI/MRI	Experimental (crossover)	VG vs. Neutral stimuli: ▴ IFG (left) ▴ PHG (left) ▴ Parietal lobe (bilateral) ▴ Thalamus (bilateral) ▴ Cerebellum (right)
									VG training amount: ▴ Medial frontal lobe (right) ▴ PrCG (bilateral) ▴ PoCG (right) ▴ PHG (right) ▴ PCu (left)
Havranek et al., 2012	2012	20	23.5 ± 3.83	Healthy young adults	VGP (>11.7 h/week) Non-VGP (0.2 h/week)	Role-playing	EEG	Experimental (factorial)	1st person view vs. 3rd person view: ▾ Parietal alpha ▾ Occipital alpha ▾ Limbic cortex alpha Active VGP vs. Passive VGP: ▴ Frontal theta
Klasen et al., 2012^*^	2012	13	(18–26)	Healthy young adults, male	VGP (15.1 ± 9.0 h/week)	Action, First Person Shooter	fMRI	Experimental (crossover)	Success vs. failure events: ▴ Head of the CN ▴ NAcc ▴ Putamen ▴ Cerebellum ▴ Thalamus ▴ SPG ▴ Motor and premotor areas High vs low focus (presence of enemies): ▴ Cerebellum ▴ Visual areas ▴ PCu ▴ Premotor areas ▾ IPS (bilateral) ▾ OFC ▾ rACC Goal-oriented vs exploratory: ▾ IPS (bilateral) ▴ FFA ▾ dACC ▾ PCu High vs Low control: ▴ Visual areas ▴ Cerebellum ▴ Thalamus ▴ Motor areas ▾ Temporal poles (bilateral) ▾ AG (bilateral)
Liu et al., 2012	2012	68	19.7 ± 2.0	Healthy young adults	–	Racing	NIRS	Experimental (randomized)	Extrinsic orders & Intrinsic orders vs. Control: ▴ Prefrontal activation Extrinsic orders vs. Intrinsic orders: ▴ Prefrontal activation (globally) ▾ Prefrontal activation (in subsequent VG trials)
Mathewson et al., 2012	2012	39	(18–28)	Healthy young adults	Low or Non-VGP (<3 h/week)	Action, shooter	EEG (ERSP)	Experimental (crossover)	Learning rate predicted by: ▴ Frontal alpha power ▴ Alpha ERSPs ▴ Delta ERSPs
Prakash et al., 2012	2012	66	22 ± 2.90 (Fixed emphasis training) 20.86 ± 2.19 (Hybrid variable-priority training) 21.48 ± 2.71 (Control)	Healthy young adults	Low or Non-VGP (<4 h/week)	Action, shooter	fMRI	Experimental (randomized)	Post vs. Pre (all groups): ▾ MFG (right) ▾ SFG (right) ▾ vmPFC HVT vs. Control: ▾ MFG (right) ▾ SFG (right)
									▾ vmPFC HVT vs. FET: ▾ MFG (right) ▾ SFG (right) ▾ vmPFC ▾ Motor cortices ▾ Sensory cortices ▾ Posteriomedial cortex
Subhani et al., 2012^**^	2012	10	(19–25)	Healthy young adults	–	Racing	EEG	Quasi-experimental (with pretest)	Gaming vs. rest: ▴ Global theta Fz/alpha Pz ratio
Voss et al., 2012	2012	29	22.24 ± 2.90	Healthy young adults	Low or Non-VGP (<3 h/week)	Action, shooter	fMRI	Experimental (randomized)	Post vs. Pre-training (FC): Changes in the DMN
Wu et al., 2012	2012	16	21.3 (Experimental) 22 (Control)	Healthy young adults	Non-VGP	Action, First Person Shooter	EEG (ERP)	Experimental (randomized with pretest)	FPS vs. Non-action: = N100 amplitude = P100 amplitude ▴ P200 amplitude ▴ P300 amplitude
Anguera et al., 2013	2013	46	67.1 ± 64.2	Healthy older adults	Low or Non-VGP (<2 h/month)	Racing	EEG	Experimental (crossover)	After VG training: ▴ Midline frontal theta power ▴ Frontal-posterior theta coherence
Bailey and West, 2013^**^	2013	31	20.40 ± 2.01 (Action) 21.77 ± 4.02 (Non-action) 24.22 ± 8.43 (Control)	Healthy adults	Non-VGP (0 h/week)	Action, First Person Shooter Puzzle, Brain Training	EEG (ERP)	Experimental (randomized)	After VG training: Action VG vs. Control: ▴ Frontal amplitude (right) ▴ Posterior amplitude (right) Non-action VG vs. Control: ▴ N200 amplitude ▴ P300 amplitude ▾ Sustained modulation centralparietal region (left) ▴ Sustained modulation frontal region Post vs Pre: Action & Non-action VG vs. Control: ▴ P300 amplitude
Berta et al., 2013	2013	22	26.3 ± 5.5	Healthy young adults	VGP and Non-VGP	Action, shooter	EEG	Experimental (crossover)	VG difficulty differences in: Alpha frequency Low-beta frequency Mid beta frequency
Khairuddin et al., 2013^*^	2013	29	21.73 ± 1.59	Healthy young adults	–	Racing	EEG	Experimental (crossover)	3D vs. 2D VG play: ▴ Occipital CPEI complexity ▴ Occipital Hjorth complexity ▴ Temporal Hjorth complexity
Krishnan et al., 2013	2013	24	–	–	Action VGP (9 h/week) Non-action VGP (15 h/week)	Action, First Person Shooter Role-playing	EEG (SSVEP)	Quasi-experimental (with control group)	Non-action VGP: (Hit rate at attended 8.6 Hz flicker) ▴ Parietal activation (task difficulty at attended 8.6 flicker) ▴ Frontal activation Action VGP: (Hit rate at ignored 3 Hz flicker) ▴ Parietal activation (task difficulty at ignored 3 Hz flicker) ▴ Frontal activation
Mathiak et al., 2013	2013	13	(18–26)	Healthy young adults, male	VGP (>5 h/week)	Action, First Person Shooter	fMRI	Quasi-experimental (crossover)	Decrease of positive affect: ▴ Insula (bilateral) ▴ Amygdala (bilateral) Increase of negative affect: ▾ vmPFC (bilateral) ▾ PCu ▾ HC
Martínez et al., 2013	2013	20	18.95 ± 2.65	Healthy young adults, female	Low or Non-VGP	Puzzle, Brain training	fMRI	Experimental (randomized, with pretest)	Post vs. pre-training (resting state): ▴ Parietofrontal correlated activity VG training vs. control group (resting state): ▴ PCu (bilateral) ▴ PCC ▴ Retrosplenial cortex ▴ Inferior parietal/supramarginal (B40) ▴ TPJ ▴ TO junction ▴ PTC (BA21, 22) ▴ Temporal pole (left) ▴ IFG (left) ▴ dlPFC & vmPFC (BA10, 11) (bilateral) ▴ MFG (BA9) (left) ▴ ACC (BA24, 32) ▴ Cuneus (BA18, 19) (bilateral) ▴ Cerebellum (bilateral) ▴ Thalamus
McGarry et al., 2013	2013	7	(60–85)	Healthy older adults	–	Strategy	fMRI	Quasi-experimental (with pretest)	After VG training (FC): ▴ PPC & AG
Tachtsidis and Papaioannou, 2013^**^	2013	30	24.00	Healthy young adults	≪Some≫ experience in VG	Action, fighting Puzzle	NIRS	Experimental (randomized with pretest)	VG playing vs. baseline: ▴ PFC Fighting vs. puzzle game: ▴ PFC (1st third of gameplay) ▾ PFC (3rd third of gameplay)
Hahn et al., 2014	2014	27	25.5 ± 4.18 (VGP) 24.5 ± 2.85 (Non-VGP)	Healthy young adults	VGP (>4 h/week) Non-VGP (0 h/week)	Role-playing, MMORPG	fMRI	Quasi-experimental (with control group)	VGP vs. Non-VGP (reward anticipation): ▾ VS VGP vs. Non-VGP (resting-state): ▴ VS regional homogeneity (right)
Nikolaidis et al., 2014	2014	45	21.74 ± 5.09	Healthy young adults	Low or Non-VGP (<4 h/week)	Action, shooter	fMRI	Experimental (crossover)	Post vs. Pre-training (predictors of working memory performance): ▴ Superior parietal lobule ▴ PoCG ▴ PCu
Strenziok et al., 2014	2014	46	69.21 ± 4.9	Healthy older adults	–	Action, Shooter real time strategy puzzle, Brain training	fMRI	Experimental (randomized with pretest)	Puzzle & Shooter vs. Strategy (FC): ▾ SPG & ITG
Yoshida et al., 2014^*^	2014	20	22.3 ± 1.2	Healthy young adults	–	Puzzle	NIRS	Experimental (crossover)	Flow vs. boredom condition: ▴ vlPFC (bilateral) ▴ dlPFC (bilateral) ▴ Frontal pole areas (bilateral)
Anderson et al., 2015	2015	40	24.0	Healthy young adults	–	Action, shooter	fMRI	Experimental (crossover)	Predictor of VG skill: ▴ DS (right) ▴ Sequential structure of whole brain activation
Hsu et al., 2015	2015	41	26.3	Healthy young adults	–	Racing	tDCS	Experimental (crossover)	Anodal tDCS vs. Sham: ▴ dlPFC (left) enhanced multitasking performance 2nd session vs. 1st session: ▴ dlPFC (left) decreased multitasking cost
Kim Y. H. et al., 2015^*^	2015	31	29.0 ± 4.1	Healthy young adults	VGP (>3 h/week) Non-VGP (<10 h/year)	Strategy	fMRI	Quasi-experimental (with control group and pretest)	VGP vs. Non-VGP: ▴ IFG (right) ▴ ACC ▴ Striatum
Liu T. et al., 2015	2015	51	21.0 ± 2.2	Healthy young adults	Low and High VGP	Racing	NIRS	Experimental (factorial)	Single vs. Paired (low VGP group) ▴ PFC Low vs. High VGP (paired group) ▾ PFC
Lorenz et al., 2015	2015	50	23.8 ± 3.9 (Experimental) 23.4 ± 3.7 (Control)	Healthy young adults	Low or Non-VGP (0.7 ± 1.97 h/month)	Action, 3D Platforms	fMRI	Experimental (randomized)	Post vs. Pre-test (reward anticipation, VG training & control group): ▾ VS Post vs. Pre-test (VG training group): = VS Post vs. Pre-test (control group) ▾ VS
McMahan et al., 2015	2015	30	20.87 (18–43)	Healthy adults	Low and High VGP (20% >20 h/week)	Action, 2D Platforms	EEG	Experimental (crossover)	High vs. Low intensity VG events: ▴ Betta power ▴ Gamma power
Patten et al., 2015	2015	–	–	–	Low or Non-VGP VGP	–	EEG (ERP)	Quasi-experimental (with control group)	VGP vs. Non-VGP: ▾ Latency Pd component
West et al., 2015	2015	59	23.88 ± 3.94 (Action) 24.36 ± 3.68 (Non-action)	Healthy young adults	Action VGP (17.9 ± 10.44 h/week) Non-Action VGP (0 h/week)	Action, First Person Shooter, Adventure	EEG (ERP)	Quasi-experimental (with control group)	Action vs. Non-VGP: ▾ Visual cortex amplitude (N2pc) in near condition. ▴ Visual cortex amplitude (N2pc) in far condition ▴ P3 component amplitude in targets.

ACC, Anterior cingulate cortex; AG, Angular gyrus; CN, Caudate nucleus; CPEI, Composite permutation entropy index; dACC, Dorsal anterior cingulate cortex; dlPFC, Dorsolateral prefrontal cortex; DS, Dorsal striatum; EEG, Electroencephalography; ERP, Event-related potentials; ERSP, Event-related spectral dynamics; FFA, Fusiform face area; FC, Functional connectivity; FG, Fusiform gyrus; fMRI, Functional magnetic resonance imaging; FPN, Frontoparietal network; HC, Hippocampus; IFG, Inferior frontal gyrus; ITG, Inferior temporal gyrus; IPS, Intraparietal sulcus; liPFC, Lateral inferior prefrontal cortex; MCA, Middle cerebral artery; MCG, Middle cingulate gyrus; MFG, Middle frontal gyrus; MRI, Magnetic resonance imaging; NAcc, Nucleus accumbens; NIRS, Near-infrarred spectroscopy; OFC, Orbitofrontal cortex; PCA, Posterior cerebral artery; PPC, Posterior cingulate cortex; PCu, Precuneus; PFC: Prefrontal cortex; PHG, Parahippocampal gyrus; PoCG, Post central gyrus; POT, Parieto-occipito-temporal; PPC, Posterior parietal cortex; PrCG, Pre-central gyrus; PTC, Posterior temporal cortex; rACC, Rostral anterior cingulate cortex; SFG, Superior frontal gyrus; SPG, Superior parietal gyrus; SSVEP, Steady state visually evoked potential; tDCS, Transcranial direct current stimulation; TO, Temporo-occipital; TPJ, Temporo-parietal junction; VG, Video game; VGP, Video game player; vmPFC, Ventromedial prefrontal cortex; VS, Ventral striatum. Articles marked with an asterisk

(*) discuss cognitive implications without directly assessing this dimension. Articles marked with a double asterisk

(**) *did not provide either empirical cognitive data nor discuss cognitive implications. The rest of the articles (non-marked) have measured cognitive correlates with specific tasks*.

**Table 6:** **Studies providing functional data dealing with VG experts or excessive gaming**.

**Ref**.	**Year**	***N***	**Mean age (range)**	**Sample**	**VG experience/Addiction**	**VG genre**	**Technique**	**Design**	**Neural correlates**
Thalemann et al., 2007^*^	2007	30	Young adults, male	28.75 ± 6.11 (Excessive VGP) 25.73 ± 8.14 (Control)	Excessive VGP (4.31 ± 2.17 h/day) Casual VGP (0.25 ± 0.46 h/day	–	EEG (ERP)	Quasi-experimental (with control group)	Excessive vs. Casual VGP (exposition to gaming cues): ▴ Pz ▴ P4
Allison and Polich, 2008^**^	2008	14	23.5 ± 5.1	Healthy young adults	Expert VGP (>10 h/week)	Action, First Person Shooter	EEG (ERP)	Experimental (crossover)	Correlated with gaming workload: ▾ N100 amplitudes (largest in Cz, smallest in Pz) ▾ P200 amplitudes (largest in Cz) ▾ N200 amplitudes (largest in Cz) ▾ sP300 amplitudes (largest in Pz, smallest on Fz)
Ko et al., 2009^*^	2009	20	(21–25)	Young adults, male	IGD (>30 h/week) Control (<2 h/day)	Role playing, MMORPG	fMRI	Quasi-experimental (with control group)	IGD vs. Control (exposition to gaming pictures): ▴ OFC (right) ▴ NAcc (right) ▴ ACC & Medial frontal cortex (bilateral) ▴ dlPFC (right) ▴ CN (right)
Granek et al., 2010^*^	2010	26	24 ± 3.1 and 26 ± 4.6	Healthy young adults, male	Expert VGP (12.8 ± 8.6 h/week) Non-VGP	–	fMRI	Quasi-experimental	Expert VGP vs. Non-VGP: ▴ SFG ▴ dlPFC (including MFG (BA 46) & IFG) ▴ vlPFC (including IFG (BA 45), ventro-orbital frontal gyrus (BA 47) and rostral lateral sulcus (BA 45) ▴ PPC (including parietooccipital sulcus (BA 7, 19), PCu (BA 7), IPS (BA 7) and IPC (BA 7)
Liu et al., 2010^*^	2010	38	Young adults	21.0 ± 1.3 (IAD) 20.0 ± 1.8 (Control)	IAD (= 6 h/day) Non-IAD	–	fMRI	Quasi-experimental (with control group)	IAD vs. Control (Regional homogeneity): ▴ Cerebellum ▴ Brainstem ▴ CG (right) ▴ PHG (bilateral) ▴ Frontal lobe (rectal gyrus, IFG & MFG) (right) ▴ SFG (left) ▴ PCu (left) ▴ PoCG (right)
						–			▴ MOG (right) ▴ ITG (right) ▴ STG (left) ▴ MTG
Doty et al., 2011^*^	2011	14	–	–	VG Dependent VG Non-dependent	Action, First Person Shooter	EEG (ERP)	Quasi-experimental (with control group)	Dependent vs. Non-dependent group: ▾ PFC (Pre-event) ▾ PFC Theta waves (Post-event) ▾ Prefrontal and frontal regions (general activation)
Dong et al., 2012^*^	2012	29	24.2 ± 3.5 (IAD) 24.6 ± 3.8 (Control)	Young adults, male	IAD (>80% time playing VG) Control (16.3 ± 4.3 Young's scale score)	–	fMRI	Quasi-experimental (with control group)	IGD vs. Control (Regional Homogeneity): ▴ Brainstem ▴ IPC ▴ Posterior cerebellum (left) ▴ MFG (left) ▾ ITG (left) ▾ Occipital lobe (left) ▾ PoCG (left) ▾ MCG (left)
Ding et al., 2013^*^	2013	41	16.94 ± 2.73 (Internet addiction group) 15.87 ± 2.69 (Control group)	Adolescents	Internet Addiction (26.44 ± 21.47 h/week; CIAS 64.59 ± 6.43) Control (10.50 ± 11.60 h/week; CIAS 45.70 ± 7.81)	–	fMRI	Quasi-experimental (with control group)	IGD vs. Control (FC): ▴ Bilateral cerebellum posterior lobe & MTG ▾ IPC (bilateral) & ITG (right)
Feng et al., 2013^*^	2013	33	16.93 ± 2.34 (IGD) 16.33 ± 2.61 (Control)	Adolescents	IGD (25.47 ± 17.89 h/week; 66.73 ± 3.01 Chen Internet Addiction Scale) Control (9.28 ± 12.90 h/week; 40.50 ± 8.42 Chen Internet Addiction Scale)	–	fMRI	Quasi-experimental (with control group)	IGD vs. Control: ▴ ITC/FG (left) ▴ PHG/Amygdala (left) ▴ Medial frontal lobe /ACC (right) ▴ Insula (bilateral) ▴ MTG (right) ▴ PrCG (right) ▴ SMA (left) ▴ CG (left) ▴ IPC (right) ▾ MTG (left) ▾ MOG (left) ▾ CG (right)
Kätsyri et al., 2013a^*^	2013	11	25.6 (22–33)	Healthy young adults, male	VG Experts (>10 h/week)	Action, First Person Shooter	fMRI	Experimental (crossover)	Winning vs. Losing: ▴ omPFC Active vs. Vicarious playing: ▾ Midbrain ▾ Striatum (especially anterior putamen, in loss events)
Kätsyri et al., 2013b^*^	2013	17	24.8 (20–33)	Healthy young adults, male	VG Experts (>10 h/week)	Action, First Person Shooter	fMRi	Experimental (crossover)	Winning vs. Losing: ▴ vmPFC ▴ VS ▴ DS Winning condition (FC): ▴ VS & ▴ Insula (right) ▴ VS & ▴ DS ▴ VS & ▴ PrCG & PoCG ▴ VS & ▴ Visual association cortices Human vs. Computer opponent: ▴ vmPFC ▴ DS
Ko et al., 2013^*^	2013	45	24.67 ± 3.11 (IGD) 24.80 ± 2.68 (Remission) 24.47 ± 2.83 (Control)	Young adults, male	IGD IGD in remission Control (Non-IGD)	Role playing, MMORPG	fMRI	Quasi-experimental (with control group)	IGD vs. Control (exposition to gaming cues): ▴ dlPFC (bilateral) ▴ PCu ▴ PHG (left) ▴ PCC ▴ ACC (right)
Kim et al., 2013	2013	5	18 ± 0	Adolescents, male	IGD (>4 h/day) Control (0 h/day)	–	EEG (ERP)	Quasi-experimental (with control group and pretest)	Pre vs. Post course: ▴ P300 fronto-central areas (bilateral) IGD vs. Control (current density, post-course): ▾ Midline paracentral lobule ▾ PCu
Latham et al., 2013	2013	31	23.27 ± 0.88 (VGP) 25.69 ± 1.19 (Non-VGP)	Healthy young adults, male	Expert VGP (34.67 ± 5.01 h/week) Non-VGP	Action, shooter; Strategy or Role Playing, MMORPG	EEG (ERP)	Quasi-experimental	Expert VPG vs. Low VPG: Earlier Visual N1
Song et al., 2013^*^	2013	39	Healthy young adults, male	20.5 ± 1.6 (2D) 20.4 ± 2.1 (3D)	Professional VGP (2D strategy VG) Professional VGP (3D strategy VG)	Real time strategy	fMRI	Quasi-experimental (with control group)	2D vs. 3D strategy game: ▴ SFG (right) ▴ Medial frontal gyrus (bilateral) ▴ Occipital lobe (right) ▾ MFG (left) ▾ FG (left) ▾ Cerebellum (left)
Yuan et al., 2013b	2013	36	Adolescents and young adults	19.4 ± 3.1 (IGD) 19.5 ± 2.8 (Control)		Role playing, MMORPG	fMRI	Quasi-experimental (with control group)	IGD vs. Control: ▴ medial OFC (left) ▴ PCu (left) ▴ SMA (left) ▴ PHG (right) ▴ MCG (bilateral)
Chen et al., 2014	2014	30	24.67 ± 3.12 (IGD) 24.47 ± 2.83 (Control)	Young adults, male	IGD (>4 h/day; 76.00 ± 12.09 Chen Internet Addiction Scale) Control (26.0 ± 0.0 Chen Internet Addiction Scale)	Role playing, MMORPG	fMRI	Quasi-experimental (with control group)	IGD vs. Control: ▾ SMA (right) ▾ preSMA (right)
Tian et al., 2014^*^	2014	12	Young adults, male	23.5 ± 2.58 (IGD) 22.7 ± 1.27 (Control)	IGD (77.6 ± 6.8 Young's scale score) Control (28.7 ± 3.7 Young's scale score)	Role playing, MMORPG	PET	Quasi-experimental (with control group and pretest)	IGD vs. Control (glucose metabolism): ▴ Cuneus (right) ▴ Calcarine (right) ▾ Medial temporal cortex (left) Within group (monoamine receptors): ▴ Correlation with IGD severity ▾ Putamen ▾ OFC/cerebellum ratio Glucose vs. monoamine: ▴ Striatum/cerebellum ratio ▴ OFC/cerebellum ratio
Wee et al., 2014^*^	2014	33	Adolescents	17.3 ± 2.6 (IAD) 17.7 ± 2.5 (Control)	IAD (4.8 ± 22 h/day; 62.4 ± 17.1 Young's Internet Addiction Scale) Control (1.3 ± 0.6 h/day; 37.0 ± 10.6 Young's Internet Addiction Scale)	–	fMRI	Quasi-experimental (with control group)	IAD vs. Control (FC): Frontal, occipital, and parietal lobes
Xing et al., 2014	2014	34	Young adults	19.1 ± 0.7 (IGD) 19.8 ± 1.3 (Control)	IGD (9.5 ± 1.3 h/day; 65.7 ± 11.6 IAT) Control (2.2 ± 1.4 h/day; 29.2 ± 4.5 IAT)	Action, Real time strategy	fMRI	Quasi-experimental (with control group)	IGD vs. Control (FC): = FC
Gong et al., 2015^*^	2015	57	23.26 ± 0.4 (Experts) 22.36 ± 0.38 (Amateurs)	Healthy young adults	Expert VGP (46.67 ± 2.1 h/week) Amateur VGP (14.2 ± 1.1 h/week)	Action, Real time strategy	fMRI	Quasi-experimental (with control group)	Experts vs. Amateurs (FC): ▴ Between insular regions (especially left) VG experience (FC): ▴ Insular functional integration (left)
Han et al., 2015	2015	47	15.2 ± 1.9 (IGD) 14.9 ± 1.9 (Control)	Adolescents, male	IGD (>30 h/week) Non-IGD	Role playing Real time strategy First Person Shooter Others	fMRI	Quasi-experimental (with control group)	IGD vs. Control: ▴ FEF (left) to dACC ▴ FEF (left) to anterior insula (right) ▴ dlPFC (left) to TPJ (left) ▴ dlPFC (right) to TPJ (right) ▴ Auditory cortex (right) to motor cortex (right) ▴ Auditory cortex (right) to SMA ▴ Auditory cortex (right) to dACC
Hong et al., 2015^*^	2015	23	13.4 ± 2.31(IGD) 14.81 ± 0.87(Control)	Adolescents, male	IGD (14.25 ±1 2.12 h/week; 57.00 ± 17.39 Young Internet Addiction Test) Controls (16.86 ± 6.51 h/week; 38.36 ± 7.31 Young Internet Addiction Test)	–	fMRI	Quasi-experimental (with control-group)	IGD vs. Control (FC): ▾ Dorsal putamen & (Posterior insula, parietal operculum) Internet addiction scores (FC): ▴ Dorsal putamen & PoCG (bilateral)
Kim H. et al., 2015^*^	2015	31	21.63 ± 5.92 (IGD) 25.4 ± 5.29 (Control)	Young adults, male	IGD (5.95 ± 2.27 h/day; 75.81 ± 4.72 Young's Internet Addiction Test) Control (<2 h/day; 23.80 ± 14.81 Young's Internet Addiction Test)	–	fMRI	Quasi-experimental (with control group)	IGD vs. Control (Regional Homogeneity): ▴ PCC ▾ STG (right) Internet addiction scores correlated in (Regional Homogeneity): ▴ Medial frontal cortex ▴ PCu/PCC ▴ ITC (left)
Lee et al., 2015	2015	38	Adolescents, male	13.6 ± 0.9 (IGD) 13.4 ± 1.0 (Control)	IGD Control (Non-IGD)	–	fMRI	Quasi-experimental (with control group)	IGD vs. Control (Angry facial stimuli): ▾ dACC ▴ Insula ▾ PPC ▴ FG IGD vs. Control (FC): ▴ (Insula & ▾ dlPFC, ▾ MTG, ▾ cerebellum, PPC)
Liu J. et al., 2015^*^	2015	40	Young adults	21.4 ± 1.0 (IGD) 20.8 ± 1.1 (Control)	IGD (= 6 h/day in internet) Control (<3 h/day in internet)	Role playing, MMORPG	fMRI	Quasi-experimental (with control group)	IGD vs. Control: ▴ SPG (right) ▴ Insular lobe (right) ▴ PCu (right) ▴ CG (right) ▴ STG (right) ▴ Brainstem (left) ▴ Frontal cortex
Luijten et al., 2015	2015	45	Young adults, male	20.83 ± 3.05 (IGD) 21.38 ± 3.03 (Control)	IGD (Video game Addiction Test score = 2.5) Control (Video game Addiction Test score = 1.5)	–	fMRI	Quasi-experimental (with control group)	IGD vs. Control: ▾ IFG (left) ▾ IPC (right)
Wang et al., 2015^*^	2015	31	Adolescents	16.94 ± 2.73 (IGD) 15.87 ± 2.69 (Control)	IGD (64.59 ± 6.43 Chen Internet Addiction Scale) Control (45.70 ± 7.81 Chen Internet Addiction Scale)	–	fMRI	Quasi-experimental (with control group)	IGD vs. Control (FC): ▾ SFG (orbital part) (bilateral) ▾ IFG (orbital part) ▾ MFG ▾ SFG
Dong and Potenza, 2016	2016	36	21.33 ± 2.18 (IGD) 21.90 ± 2.33 (Control)	Young adults, male	IGD (>80% online time) Low or Non-VGP	–	fMRI	Quasi-experimental (with control group)	IGD vs. Control: ▾ ACC ▾ PCC ▾ MTG ▾ IFG (RT) ▾ STG (RT)
Gong et al., 2016	2016	45	23.3 ± 4.3 (Experts) 22.3 ± 3.46 (Amateurs)	Healthy young adults, male	Experts (ELO > 1,800) Amateurs (ELO <1,200)	Action, Real time strategy	fMRI	Quasi-experimental (with control group)	Action VG Experts vs. Amateurs: ▴ SN & CEN (global characteristics) ▴ Local regions of SN & CEN (nodal characteristics) ▴ SN & CEN (FC)
Han et al., 2016	2016	102	20.2 ± 3.2 (IGD) 20.2 ± 2.9 (Control)	Adolescent or young adults, male	IGD (>30 h/week) Low or Non-VGP	Role playing Real time strategy First Person Shooter Others	fMRI	Quasi-experimental (with control group)	IGD vs. Control (During executive task): ▴ Lateral occipital cortex (right) ▴ PCu
Jin et al., 2016^*^	2016	46	19.12 ± 1.05 (IGD) 18.76 ± 1.81 (Control)	Adolescents	IGD (5.32 ± 2.10 h/day) Low or Non-VGP (2.07 ± 1.39 h/day)	Action, Real time strategy	fMRI/MRI	Quasi-experimental (with control group)	IGD vs. Control (FC): ▾ (dlPFC, OFC, ACC & SMA) & (Insula, temporal cortices, occipital cortices) ▾ (dlPFC, OFC, ACC & SMA) & (DS, pallidum, thalamus)
Park et al., 2016	2016	36	Young adults, male	24.2 ± 3.2 (OGA-CBT) 23.6 ± 2.7 (OGA-VRT) 23.3 ± 2.9 (Control)	IGD (>30 h/week) Control (<3 h/week)	–	fMRI	Quasi-experimental (with control group)	IGD vs. Control (baseline, ALFF): ▾ MFG (right) IGD vs. Control (baseline, FC) ▾ Cortico-striatal limbic circuit
Zhang et al., 2016	2016	115	Young adults	22.28 ± 1.98 (IGD) 23.02 ± 2.09 (Control)	IGD (78.36 ± 8.43 Chen Internet Addiction Scale) Control (43.49 ± 9.64 Chen Internet Addiction Scale)	–	fMRI	Quasi-experimental (with control group)	IGD vs. Control (FC): ▴ (Anterior insula & ACC, Putamen, Angular gyrus, PCu) ▴ (Posterior insula & PoCG, PrCG, SMA, STG) IGD severity (FC): ▴ (Anterior insula & Angular gyrus, STG) ▴ (Posterior insula & STG)

ACC, Anterior cingulate cortex; CEN, Central executive network; CG, Cingulate gyrus; CIAS, Chen's Internet addiction scale; CN, Caudate nucleus; dACC, Dorsal anterior cingulate cortex; dlPFC, Dorsolateral prefrontal cortex; DS, Dorsal striatum; FC, Functional connectivity; FEF, Frontal eye fields; FG, Fusiform gyrus; IFG, Inferior frontal gyrus; IGD, Internet gaming disorder; IPC, Inferior parietal cortex; IPS, Intraparietal sulcus; ITC, Inferior temporal cortex; ITG, Inferior temporal gyrus; MCG, Middle cingulate gyrus; MFG, Middle frontal gyrus; MOG, Middle occipital gyrus; MTG, Middle temporal gyrus; NAcc, Nucleus accumbens; OFC, Orbitofrontal cortex; omPFC, Orbitomedial prefrontal cortex; PCC, Posterior cingulate cortex; PCu, Precuneus; PFC, Prefrontal cortex; PHG, Parahippocampal gyrus; PoCG, Post-central gyrus; PrCG, Pre-central gyrus; preSMA, Pre-supplementary motor area; SFG, Superior frontal gyrus; SMA, Supplementary motor area; SN, Salience network; SPG, Superior temporal gyrus; STG, Superior temporal gyrus; TPJ, Temporo-parietal junction; vlPFC, Ventrolateral prefrontal cortex; VS, Ventral striatum. Articles marked with an asterisk

(*) discuss cognitive implications without directly assessing this dimension. Articles marked with a double asterisk

(**) *did not provide either empirical cognitive data nor discuss cognitive implications. The rest of the articles (non-marked) have measured cognitive correlates with specific tasks*.

**Table 7:** **Studies providing functional data focused on the violent contents of VG**.

**Ref**.	**Year**	***N***	**Sample**	**Age**	**VG experience**	**VG genre**	**Technique**	**Design**	**Neural correlates**
Bartholow et al., 2006	2006	39	Healthy young adults	19.50	Violent VGP Non-violent VGP	“Violent” VG “Non-violent” VG	EEG (ERP)	Quasi experimental (with control group)	Violent vs. Non-violent: ▾ P300 amplitudes
Mathiak and Weber, 2006^*^	2006	13	Healthy young adults, male	(18–26)	VGP (15.1 ± 9.0 h/week)	Action, First Person Shooter	fMRI	Quasi-experimental	During violent scenes: ▴ dACC ▾ rACC ▾ Amygdala
Weber et al., 2006^*^	2006	13	Healthy young adults	23.00 (18–26)	VGP (15.1 h/week)	Action, First Person Shooter	fMRI	Experimental (crossover)	During virtual violence exposure: ▾ Amygdala ▾ ACC
Wang et al., 2009	2009	44	Healthy adolescents	14.8 ± 1.2 (Violent group) 15.0 ± 1.1 (Non-violent group)	–	Sports, Racing Action, First person shooter	fMRI	Experimental (randomized)	Violent vs. Non-violent (Counting Stroop task): ▾ PFC Violent vs. Non-violent (Counting Stroop task, FC): ▾ dlPFC & ACC (left) Violent vs. Non-violent (Emotional Stroop task): ▴ Amygdala (right) ▾ Medial PFC Violent vs. Non-violent (Emotional Stroop task, FC): ▾ Amygdala & Medial PFC (non-violent group) = FC (violent group)
Hummer et al., 2010	2010	45	Healthy adolescents	14.9 ± 0.3 (Non-violent) 14.5 ± 0.3 (Violent)	–	Sports, Racing Action, First Person Shooter	fMRI	Experimental (randomized)	Violent vs. Non-violent (Go/No-go): ▾ dlPFC (right) Non-violent VG (FC): ▾ dlPFC & ▴ PCu
Regenbogen et al., 2010^*^	2010	22	Healthy young adults, male	25.9 ± 2.9	VGP (131 h/year violent VG) Low or Non-VGP (6 h/year violent VG)	Violent VG	fMRI	Quasi-experimental (with control group)	Real vs. Non-violent content (VGP): ▴ IFG (right) ▴ Lingual gyrus (left) ▴ STG Virtual vs. Non-violent content (VGP): ▴ IFG (bilateral) ▴ Occipital cortex ▴ PoCG ▴ MTG (right) ▴ FG (left) Real vs. Non-violent content (Control): ▴ Frontal regions (left) ▴ Insula ▴ SFG Virtual vs. Non-violent content (Control): ▴ Posterior regions
Bailey et al., 2011^*^	2011	48	Healthy young adults, male	19.73 ± 1.28 (low VGP) 19.87 ± 3.52 (high VGP)	Low VGP (3 h/week) High VGP (33 h/week)	“Violent” VG	EEG (ERP)	Quasi-experimental (with control group)	High vs. Low VGP: (Negative & violent) vs. (neutral and positive) stimuli: ▾ FCz (@125 ms) ▾ Oz (@280 ms) Early posterior negativity (EPN) ▴ PO4 & F9 (500-1000 ms) Late positive potential (LPP) High (positive & violent vs. neutral stimuli) vs. Low VGP (Positive vs. neutral stimuli): ▾ Fpz (@100 ms) ▴ C3, CP3 (400–900 ms) ▾ Iz (400–900 ms) Low vs. High VGP (negative vs. violent stimuli): ▴ F9 (sustained) ▴ TP8 (sustained) ▴ PO9 (@300 ms)
Engelhardt et al., 2011	2011	70	Healthy young adults	(18–22)	Violent VGP Non-violent VGP	Action, First Person Shooter, Adventure Action, Adventure, Sports, Platforms	EEG (ERP)	Quasi-experimental (with control group)	Violent vs. Non-violent: ▾ P3 component at parietal (P3, Pz, and P4) regions
Mathiak et al., 2011^*^	2011	13	Healthy young adults, male	22.7 ± 2.0	VGP (15.1 ± 9.0 h/week)	Action, First Person Shooter	fMRI	Quasi-experimental	(Failure & success events) vs. Baseline: ▴ Visual cortex Failure vs. Success events: ▾ OFC ▾ CN Negative vs. Positive affect (only failure events): ▾ Temporal pole (right)
Montag et al., 2012^*^	2012	40	Healthy young adults, male	23.33 ± 4.45	Experienced VGP in First person Shooters (18.83 ± 9.12 h/week) Low or Non-VGP (2.00 ± 3.82 h/week)	Action, First Person Shooter	fMRI	Quasi-experimental (with control group)	VGP vs. Control (during negative emotional stimuli): ▾ Lateral medial frontal lobe (left) VGP vs. Control (during VG cues): ▴ Frontal regions ▴ Temporal regions
Chou et al., 2013^*^	2013	30	Healthy young adults	24.67 ± 4.7	VGP (3.1 h/week)	Action, Fighting Action, 3D platforms	SPECT	Experimental (crossover)	Post vs. Pre-training: ▾ PFC ▴ Temporal cortex ▴ Occipital cortex Violent vs. Non-violent VG: ▾ dACC (in males)
Lianekhammy and Werner-Wilson, 2015^*^	2015	45	Healthy adolescents	14.3 ± 1.5	Brain training VG group (4.8 ± 10.6 h/week) Violent VG group (17.7 ± 17.4 h/week) Non-violent VG (9.3 ± 8.4 h/week)	Puzzle Action, First Person Shooter Action, 3D platforms	EEG	Experimental (randomized)	High empathy vs. Low empathy: ▴ Right hemisphere frontal EEG asymmetry scores (violent and non-violent) ▴ Right hemisphere High vs. Low helpfulness: ▴ Left hemisphere (non-violent group)
Liu Y. et al., 2015	2015	49	Healthy young adults	20.76 ± 1.76	–	Strategy Sports, Racing	EEG (ERP)	Experimental (randomized)	Prosocial vs. Neutral VG: ▾ P300 amplitudes
Petras et al., 2015	2015	30	Healthy adults	25.96 (18–44)	21 Non habitual VGP (< once a week) 9 Habitual VGP (= once a week)	Action, Shooter	EEG (ERP)	Experimental (randomized, factorial)	Egocentric vs. Bird-view perspective: ▴ N1 amplitudes (shoot task) ▾ Pre-stimulus alpha power (shoot task)
Zvyagintsev et al., 2016^*^	2016	18	Healthy young adults, male	25.1 ± 2.7	VGP (>5 h/week)	Sports, Racing (Violent)	fMRI	Experimental (crossover)	Violent vs. Non-violent (FC): ▾ Sensory-motor networks ▾ Reward network ▾ DMN ▾ FPN (right)

3D, Three-dimensional; ACC, Anterior cingulate cortex; CN, Caudate nucleus; dACC, Dorsal anterior cingulate cortex; dlPFC, Dorsolateral prefrontal cortex; DMN, Default mode network; EEG, Electroencephalography; ERP, Event-related potentials; FC, Functional connectivity; FG, Fusiform gyrus; fMRI, Functional Magnetic Resonance Imaging; FPN, Frontoparietal network; IFG, Inferior frontal gyrus; MTG, Middle temporal gyrus; OFC, Orbitofrontal cortex; Pcu, Precuneus; PFC, Prefrontal cortex; PoCG, Post-central gyrus; rACC, Rostral anterior cingulate cortex; SFG, Superior frontal gyrus; SPECT, Single-photon emission computed tomography; STG, Superior temporal gyrus; VG, Video game; VGP, Video game player. Articles marked with an asterisk

(*) discuss cognitive implications without directly assessing this dimension. Articles marked with a double asterisk

(**) did not provide either empirical cognitive data nor discuss cognitive implications. The rest of the articles (non-marked) have measured cognitive correlates with specific tasks.

The rich diversity of methodologies and research goals means that the study of functional brain correlates covers practically all regions of the brain. The most studied areas are found in frontal and prefrontal regions and are concerned with high-order cognitive processes and motor/premotor functions. Activity changes in parietal regions, like the posterior and superior parietal lobe, relevant for diverse functions such as sensory integration and visual and attentional processing, are also a common find. The anterior and posterior cingulate cortices, together with other limbic areas, such as the amygdala, and the entorhinal cortex, display activity changes possibly as a consequence of learning and emotion processing and memory. Structures in the basal nuclei also have a prominent role, particularly the striatum, in studies related to VG addiction. Finally, we must not overlook a series of brain regions which do not appear as frequently, such as occipital and temporal cortices, the cerebellum, the thalamus, and the hippocampus, where distinctive activity patterns have also been observed as a result of VG play.

## Discussion

Due to the given amount of data provided in the reviewed articles, we decided to categorize all the information based on the cognitive functions which are associated with the neurophysiological correlates, rather than focusing on the main research goal for each study. Thus, the discussion has been grouped into six main sections: attention, visuospatial skills, cognitive workload, cognitive control, skill acquisition, and reward processing. These cognitive processes are not clearly independent since they present some degree of overlap. This is particularly relevant in the cases of cognitive workload, which may be linked to virtually any cognitive function, and attention, which is also closely related to cognitive control, among other functions. Nevertheless, after analyzing the literature, virtually all the articles included in this review focused on one or more of the mentioned cognitive functions in order to explain their findings. Thus, the proposed categories have sufficient presence in the literature to justify their use as separate domains for the study of cognition. While they should not be understood as independent aspects of cognition, the chosen categorization will allow a link between the underlying neural correlates and corresponding behavior to be easily established.

Within each one of the sections, structural and functional correlates are discussed according to their contributions to cognitive functioning, including possible inconsistencies between studies and the presence of transfer effects. Owing to the close link between VG violence, limbic and reward systems, and the possible abnormal reward mechanisms in addicted players, studies previously classified with violence in VGs and VG addiction are predominantly discussed in the reward processing section.

### Attention

Attentional resources are one of the main cognitive domains in which VGs are involved and one of the most researched. The involvement of attentional networks during gameplay is closely related with other brain regions responsible for cognitive control, especially when more complex operations toward a specific goal are required. Many brain regions are involved in attention, particularly nodes in the dorsal frontoparietal system, mediating top-down attentional processes in goal-oriented behavior, but also nodes in the ventral network, responsible for bottom-up sensory stimulation (e.g., Vossel et al., 2014) dealing with those salient stimuli to which the player must pay attention.

There is evidence that VGPs display enhanced performance in a range of top-down attentional control areas, such as selective attention, divided attention, and sustained attention (Bavelier et al., 2012b). The ACC is an area that consistently shows functional activity during VG play due to its involvement as the main hub in top-down attentional processes (selective or focused attention) and goal-oriented behavior (e.g., Anderson et al., 2011^*^; Bavelier et al., 2012b).

Non-VGPs, compared to VGPs, showed greater frontoparietal recruitment, a source of selective attention, as task demands increased, showing that habitual gamers have more efficient top-down resource allocation during attentional demanding tasks (Bavelier et al., 2012a). That resource optimization effect can also be observed in attentional control areas, such as the right middle frontal gyrus (MFG), right superior frontal gyrus (SFG), and the ventromedial prefrontal cortex (vmPFC) (Prakash et al., 2012^*^). Functional connectivity changes in the attentional ventral stream, particularly in occipitotemporal WM, responsible for bottom-up reorienting toward novel stimuli, have also been observed as a result of VG training and were linked to cognitive improvement (Strenziok et al., 2014^*^). Integration between attentional and sensoriomotor functions has been observed in expert VGPs in the form of increased structural GM and functional connectivity in anterior and posterior insular sub regions where long-term exposure to attentional VG demands coordinated with the fine skills involved in using the VG controller may have resulted in plastic changes in these two regions that are respectively involved in attentional and sensoriomotor networks (Gong et al., 2015^*^).

Using electrophysiological techniques, it seems that VG play correlates with an increment of the frontal midline theta rhythm, associated with focused attention (Pellouchoud et al., 1999^*^), and increases with VG practice (Sheikholeslami et al., 2007^**^; Smith et al., 1999), both in an action and a puzzle VG, attributable to ACC activity. Likewise, amplitudes in the P200 (Wu et al., 2012), an early visual stimuli perceptual component, and P300 components (Mishra et al., 2011; Wu et al., 2012), which involved in early stages of decision-making, were also linked to top-down spatial selective attention improvements after training and lifetime exposure to action VG. Action VGPs and non-action VGPs seem to respond differently in the way they deploy attention to central and peripheral targets in visual attention tasks, as measured by the N2pc component (West et al., 2015), which is also linked to selective attention.

If we consider different VG genres, it seems that action VGs are better at improving selective attention than other slow-paced VGs such as role-playing games (RPG) (Krishnan et al., 2013), puzzle (Green and Bavelier, 2003), or strategy VGs (Tsai et al., 2013) which require high planning skills and other forms of proactive cognitive control. This is probably due to the extensive use of attentional systems, paired with precise timings that action VGs require. While these improved attentional skills are typically observed in habitual VGPs, it is possible to achieve long-lasting improvements as a result of a single VG training procedure (Anguera et al., 2013).

### Visuospatial skills

Visuospatial skills encompass processes that allow us to perceive, recognize, and manipulate visual stimuli, including visuomotor coordination and navigational skills, and VGs are predominantly interactive visual tasks.

The areas implicated in visuospatial processing have traditionally been classified along a visual ventral stream (responsible for object recognition) and a visual dorsal stream (responsible for spatial location). Both depart from the visual cortex, in the occipital lobe, and reach the posterior parietal cortex (dorsal stream) and the inferior temporal cortex (ventral stream). More recent proposals have refined that model, broadening the traditional conceptualization of the two-stream model (for further details see Kravitz et al., 2011). Among other nodes, the role of the hippocampus stands out for its function in higher order visual processing and memory (Kravitz et al., 2011; Lee A. C. H. et al., 2012).

Neural correlates related to visuospatial skills have been detected in relationship with structural volume enlargements of the right hippocampus (HC), both in long-term gamers and experimentally after a VG training period (Kühn et al., 2013; Kühn and Gallinat, 2014^*^). Increased hippocampal volumes were also found by Szabó et al. (2014^**^), although the authors do not attribute that effect to the VG training. The entorhinal cortex, associated with navigational skills (Schmidt-Hieber and Häusser, 2013), which together with the HC is involved in spatial memory (Miller et al., 2015), was also correlated with lifetime experience in logic/puzzle and platform VG (Kühn and Gallinat, 2014^*^).

Decreased activation in occipitoparietal regions, associated with the dorsal visuospatial stream (Goodale and Milner, 1992), has also been linked to improved visuomotor task performance, suggesting a reduction of the cognitive costs as a consequence of the VG training, dependent on the training strategy used in the VG (Lee H. et al., 2012). Earlier N100 latencies in the visual pathways are another feature found in long-term VGPs, which may contribute to faster response times in visual tasks after years of practice (Latham et al., 2013).

Reduced WM integrity in interhemispheric parietal networks for spatially-guided behavior could be another symptom for a decreased reliance on specific visuospatial networks after VG training as performance improved (Strenziok et al., 2013^*^). However, other studies found that increased WM integrity in visual and motor pathways was directly responsible for better visuomotor performance in long term VGPs (Zhang et al., 2015^*^). Despite these connectivity changes, brain functional differences between VGPs and non-VGPs do not always reflect performance in visuospatial skills, which were best predicted by non-visual areas (Kim Y. H. et al., 2015^*^).

### Cognitive workload

Brain activation patterns depend on the cognitive demands of the environment and also on the associated level of workload (Vogan et al., 2016), which is directly related to the allocation of resources to the working memory and its associated attentional processes (Barrouillet et al., 2007). When we manipulate this variable and observe its neural correlates, it is likely that we are seeing the result of neural recruitment mechanisms as the cognitive demands increase (Bavelier et al., 2012a). VGs have often been employed to obtain cerebral measures of cognitive workload, given the ability to adjust many of their features, particularly in a purpose-made VG, such as the popular Space Fortress. Due to the nature of this task, it is likely that functional changes related to the manipulation of cognitive load appear along the attentional networks and in specific key nodes related to executive functions, mainly in prefrontal and parietal cortices.

Cognitive workload is not a unitary concept; some studies have been able to identify different activation patterns by manipulating the difficulty of a task (e.g., Anderson et al., 2011^*^). Namely, the number of stimuli appearing simultaneously on the screen and the complexity of each stimulus seem to elicit different responses from the brain. For instance, in the context of an air traffic control simulator, when directly manipulating the task difficulty by increasing the number of planes that a participant had to attend, the theta band power increased (Brookings et al., 1996). Theta band power also displayed higher power compared to a resting condition, and gradually increased during gameplay (Sheikholeslami et al., 2007^**^). The theta band seems to be directly related to the level of cognitive demand in a wide range of cognitive abilities, such as attention, memory, and visuospatial processes, although this finding is not universal and decreased theta band power has been observed as a feature of sustained attention. So it appears that it is both related to task complexity and levels of arousal and fatigue. On the other hand, beta band power seemed to be more associated with the complexity of the task, especially in frontal and central areas, likely indicating a qualitative change in the cognitive strategy followed by the participant or the type of processing done by the brain (Brookings et al., 1996).

Assessing cognitive workload with ERP shows that during VG play, amplitudes tend to correlate negatively with game difficulty in expert VGPs, with most ERP (P200, N200) having its maximum amplitude in frontoparietal locations, with the exception of the P300, being larger in parietal regions (Allison and Polich, 2008). This is consistent with previous literature about cognitive workload related to attention and working memory demands and ERP peak amplitude decrements (Watter et al., 2001).

Frontoparietal activity, linked to attentional processes, also exhibits recruitment effects as game difficulty increases, which also affects reaction times, making them slower (Bavelier et al., 2012a). As mentioned above, comparing habitual VGPs with non-VGPs, it appears that the former show less recruitment of frontoparietal networks when compared to the non-gamers, which could be attributed to their VG experience and the optimization of their attentional resources (Bavelier et al., 2012a). Increased blood flow in prefrontal areas like dlPFC was also associated with increasing cognitive demands related to attention, verbal and spatial working memory and decision making (Izzetoglu et al., 2004^*^).

The intensity of the events displayed in the VG was also linked with certain electrophysiological correlates. High intensity events, such as the death of the VG character, were associated with increased beta and gamma power when compared with general gameplay (McMahan et al., 2015).

### Cognitive control

During the course of a VG, the player can encounter many situations in which he has to use one of several possible actions. For instance, while playing a game, the player might be required to interrupt and quickly implement an alternate strategy, or manipulate a number of elements in a certain way in order to solve a puzzle and progress in the storyline. All these abilities can be characterized under the “umbrella” of cognitive control, which includes reactive and proactive inhibition, task switching and working memory (Obeso et al., 2013). These cognitive control aspects are key to overcoming the obstacles found the VG. In fact, they are frequently used in parallel (Nachev et al., 2008) in order to engage in goal-directed behavior. These processes have their neural substrate in the prefrontal cortex, supported by posterior parietal areas and the basal ganglia (Alvarez and Emory, 2006). Therefore, most changes regarding cognitive control observed after VG play will likely be detected in these regions.

Indeed, prefrontal regions are one of the brain areas in which GM volumetric changes have been observed as a result of a cognitive training with a VG, which is remarkable if we consider that the common VG training period spans from a few weeks to a couple of months. These regions, such as the dlPFC, determinant for cognitive control (Smith and Jonides, 1999), show volumetric changes that seem to correlate with VG performance and experience, likely as a result of the continuous executive demands found in a VG, such as attentional control and working memory (Basak et al., 2011). These volumetric changes even result in correlations with transfer effects in cognitive control tasks (Hyun et al., 2013). Volumetric-behavioral correlations work both ways, since individuals with decreased orbitofrontal cortex (OFC) volumes as a consequence of VG addiction show poorer performance in similar tasks (Yuan et al., 2013a).

During VG play, these prefrontal regions increase their activation in response to the cognitive demands (game difficulty) and display a positive correlation with performance measures (Izzetoglu et al., 2004^*^). Still, prefrontal activity is not only affected by the complexity of the task, but also by the nature of the task and the individual differences of the participants (Biswal et al., 2010). Some research groups have found deactivation of dorsal prefrontal regions during gameplay. A possible explanation for this phenomenon could be the interference effect of attentional resources during visual stimuli, since activity in the dlPFC remained stable while passively watching a VG, but not while actively playing it (Matsuda and Hiraki, 2004^*^). Likewise, the same team also found that finger movement while handling the game controller did not seem to contribute as a source of prefrontal deactivation. Further studies also noted that the observed prefrontal deactivation was not affected by age or performance level (Matsuda and Hiraki, 2006^*^), although some authors have challenged that finding, claiming that prefrontal activation during video gaming was age-dependent, where most adults tended to show increased prefrontal activity while it was attenuated in some of the children. So prefrontal activation could be a result of age, game performance, level of interest and attention dedicated to the VG (Nagamitsu et al., 2006^**^).

It has been possible to establish a causal relationship between dlPFC activation and cognitive control using non-invasive stimulation methods. Stimulating the left dlPFC using tDCS results in a perceptible improvement in multitasking performance in a three-dimensional VG (Hsu et al., 2015).

Changes in functional activity after a training period in other executive-related nodes, such as the superior parietal lobe (SPL) have also been associated with working memory improvements (Nikolaidis et al., 2014).

Connectivity-wise, Martínez et al. (2013) found resting-state functional connectivity changes in widespread regions (frontal, parietal, and temporal areas) as a result of a VG training program, which were attributed to the interaction of cognitive control and memory retrieval and encoding.

Despite the observed structural and functional changes in prefrontal areas, executive functions trained in a VG show poor transfer effects as measured with cognitive tasks (Colom et al., 2012; Kühn et al., 2013). Others, showing neural correlates related to executive functions, visuospatial navigation and fine motor skills, failed to observe far transfer effects even after a 50 h training period, as measured by neuropsychological tests (Kühn et al., 2013). By studying lifelong experts or professional gamers, some studies have detected structural GM changes that correlated with improved executive performance, involving posterior parietal (Tanaka et al., 2013), and prefrontal (Hyun et al., 2013) regions. Regarding structural connectivity, WM integrity changes in thalamic areas correlated with improved working memory, but integrity of occipitotemporal fibers had the opposite effect (Strenziok et al., 2014). VG experience also seems to consolidate the connectivity between executive regions (dlPFC and the posterior parietal cortex -PPC-) and the salience network, composed by the anterior insula and the ACC, and responsible for bottom-up attentional processes (Gong et al., 2016).

Different VG genres seem to affect which cognitive skills will be trained. Training older adults in a strategy VG seemed to improve verbal memory span (McGarry et al., 2013), but not problem solving or working memory, while using a 2D action VG improved everyday problem solving and reasoning. Transfer effects were even more relevant in the case of a brain training/puzzle VG, where working memory improvements were also observed (Strenziok et al., 2014). Using a younger sample, working memory improvements were detected after training with a 2D action VG (Space Fortress, Nikolaidis et al., 2014). Nevertheless, training periods found in scientific literature vary greatly and it is difficult to ascertain if a lack of transferred skills cannot be due to a short training period.

Regarding electrophysiological methods, electroencephalography studies have shown functional correlations with alpha oscillations in the frontal cortex that could reflect cognitive control engagement in the training VG (Mathewson et al., 2012).

### Skill acquisition

Several studies have attempted to determine which regions could act as predictors for skill acquisition. Since this is a domain in which multiple cognitive functions are involved, volumetric and functional changes will appear in a wide range of cortical regions. Most of the learning in VGs is non-declarative, including visuospatial processing, visuomotor integration, and motor planning and execution. Improvements in these areas will generally lead to decreased cortical activation in the involved areas due to the optimization of resources, whereas this is not the case for striatal and medial prefrontal areas, which display a distinctive pattern of activation and typically increase their activity due to skill acquisition (Gobel et al., 2011).

Striatal volumes were determined as predictors for skill acquisition, although structural changes in the hippocampal formation were not (Erickson et al., 2010). Particularly, the anterior half of the dorsal striatum was the region which more accurately predicted skill acquisition in a complex VG (Vo et al., 2011). Other areas identified as predictors were the medial portion of the Brodmann area 6, located in the frontal cortex and associated with motor control in cognitive operations and response inhibition and the cerebellum, likely associated with motor skill acquisition (Basak et al., 2011). The same authors also considered the post-central gyrus, a somatosensory area that could be related to a feedback mechanism between prefrontal and motor regions, while the volume of the right central portion of the ACC also correlated with skill acquisition and is responsible for monitoring conflict. Finally, dlPFC volumes, with a central role on the executive functions, also showed correlation with VG performance over time (Basak et al., 2011).

On a functional level, Koepp et al. (1998^**^) was the first team to identify a relationship between striatum activity, associated with learning and the reward system, and performance level in a VG. The study by Anderson et al. (2015) also support the notion that the striatum, particularly the right dorsal striatum, composed of the caudate nucleus and the claustrum, is a key area in skill acquisition. However, the same team was able to predict learning rates more accurately by comparing whole sequential brain activation patterns to an artificial intelligence model.

Learning gains seemed to be best predicted by individual differences in phasic activation in those regions which had the highest tonic activation (Anderson et al., 2011^*^). Differences related to learning rates were also observed in the activation of the default mode network, especially when different training strategies were employed by the participants. Using electrophysiological methods, the best predictors were the alpha rhythms (Smith et al., 1999), particularly frontal regions, and alpha and delta ERSP, which are associated with cognitive control (task switching and inhibition) and attentional control networks (Mathewson et al., 2012). Frontal midline theta rhythms, linked with focused concentration and conscious control over attention, seemed to increase over the course of the training sessions with a VG (Smith et al., 1999).

### Reward processing

#### Addiction

VG addiction is understood as an impulse-control disorder with psychological consequences, not unlike other addictive disorders, especially non-substance addictions such as pathological gambling (Young, 1998). Internet Gaming Disorder (IGD) has been recently proposed for inclusion as a psychiatric diagnosis under the non-substance addiction category in the Diagnostic and Statistical Manual for Mental Disorders 5th ed. (DSM-5) (American Psychiatric Association, 2013), with its diagnostic criteria being adapted from those of pathological gambling. Efforts in order to find a consensus regarding its assessment are still ongoing (Petry et al., 2014). In some cases, VG addiction is included as a subset within the broader definition of Internet addiction, although this categorization is not always consistent, since many VGs in which addiction is studied do not have an online component. Several instruments have been developed to assess gaming addictions: the Internet Addiction Test (IAT) by Young (1998) and the Chen Internet Addiction Scale (CIAS) (Chen et al., 2004) being the most used in research and clinical practice.

Within the VG literature, there is a great deal of interest in knowing the neurobiological basis of VG addiction and whether it can be related to other behavioral addictions by observing abnormal reward processing patterns. This seems to be the case, since many regions involved in the reward system have been found affected in people with VG addiction (e.g., Liu et al., 2010^*^; Hou et al., 2012^*^; Hahn et al., 2014). Among the complex set of structures that are involved in the reward system, the cortico-ventral basal ganglia circuit is the center of the network responsible for assessing the possible outcomes of a given behavior, especially in those situations where, during a goal-oriented behavior, complex choices must be made and the value and risk of secondary rewards must be weighed (Haber, 2011).

Differential structural and functional changes in addicted individuals can be found throughout the reward system. The main components of this circuit are the OFC, the ACC, the ventral striatum, ventral pallidum, and midbrain dopaminergic neurons (Haber, 2011), but many other regions seem to be involved in the wider context of addiction.

By exposing the participants to gaming cues, it is possible to elicit a craving response and study which regions show stronger correlation in IGD patients compared to controls. The model proposed by Volkow et al. (2010) involves several regions, which are mentioned consistently across studies, to explain the complexity of the craving. First, the precuneus, which showed higher activation in addicted individuals (Ko et al., 2013^*^), is an area associated with attention, visual processes, and memory retrieval and integrates these components, linking visual information (the gaming cues) to internal information. Regions commonly associated with memory and emotional functions are also involved: the HC, the parahippocampus and the amygdala seem responsible for providing emotional memories and contextual information for the cues (Ding et al., 2013^*^), regions where subjects showed higher activation (O'Brien et al., 1998). Central key regions of the reward system, like the limbic system and the posterior cingulate have a role in integrating the motivational information and provide expectation and reward significance for gaming behaviors (O'Doherty, 2004). The OFC and the ACC are responsible for the desire for gaming and providing a motivational value of the cue-inducing stimuli (Heinz et al., 2009), contributing to the activation and intensity of the reward-seeking behavior (Kalivas and Volkow, 2005; Brody et al., 2007; Feng et al., 2013^*^). In the last step, prefrontal executive areas such as the dlPFC have also shown involvement during craving responses (Han et al., 2010a^*^; Ko et al., 2013^*^), and are linked to the formation of behavioral plans as a conscious anticipation of VG play. All these frontal regions[dlPFC, OFC, ACC, and the supplementary motor area (SMA)] tend to show reduced GM volumes in participants with IGD (Jin et al., 2016^*^).

Striatal volumes, particularly the ventral striatum, responsible for a key role in reward prediction, were reduced in people with excessive internet gaming compared to healthy controls (Hou et al., 2012^*^) and in the insula, with its role in conscious urges to abuse drugs (Naqvi and Bechara, 2009).

Overall, these features are characteristic of reward deficiencies that entail dysfunctions in the dopaminergic system, a shared neurobiological abnormality with other addictive disorders (Ko et al., 2009^*^, 2013^*^; Cilia et al., 2010; Park et al., 2010; Kim et al., 2011).

Several regions seem to be related to the intensity of the addiction. In a resting state paradigm, connectivity between the left SPL, including the posterior cingulate cortex (PCC), and the right precuneus, thalamus, caudate nucleus, nucleus accumbens (NAcc), SMA and lingual gyrus (regions largely associated with the reward system) correlated with the CIAS score, while at the same time, functional connectivity with the cerebellum and the superior parietal cortex (SPC) correlated negatively with that score (Ding et al., 2013^*^). The distinctive activation and connectivity patterns related to the PCC (Liu et al., 2010^*^), an important node in the DMN and reward system (Kim H. et al., 2015), could be used as a biomarker for addiction severity, both in behavioral and substance dependence. As the addiction severity increases, changing from a voluntary to a compulsive substance use, there is a transition from prefrontal to striatal control, and also from a ventral to a dorsal striatal control over behavior (Everitt and Robbins, 2005), Matching evidence in the form of weaker functional connectivity involving the dorsal-caudal putamen has been found in IGD patients (Hong et al., 2015^*^).

It is important to note that, even controlling the amount of time playing VGs, professional and expert gamers display very different neural patterns compared to addicted VGPs. Gamers falling into the addiction category show increased impulsiveness and perseverative errors that are not present in professional gamers and, on a neural level, they differ in GM volumes in the left cingulate gyrus (increased in pro-gamers) and thalamus (decreased in pro-gamers), which together may be indicative of an unbalanced reward system (Sánchez-González et al., 2005; Han et al., 2012b).

#### Exposure to violent content

Many articles use violent VGs in their designs as a way to study the effects of violence exposure, emotional regulation and long-term desensitization. Exposure to violent content has been associated with reduced dlPFC activity and interference in executive tasks (inhibition, go/no-go task) (Hummer et al., 2010), which cannot be interpreted without studying the link with the limbic and reward systems. It is likely that repeated exposure to violent content will trigger desensitization processes that affect regions linked to emotional and attentional processing, particularly a frontoparietal network encompassing the left OFC, right precuneus and bilateral inferior parietal lobes (Strenziok et al., 2011). It is hypothesized that this desensitization may result in diminished emotional responses toward violent situations, preventing empathy and lowering the threshold for non-adaptive behaviors linked to aggressiveness (Montag et al., 2012).

Limbic areas are associated with violence interactions, shown by the activation changes detected in the ACC and the amygdala in the presence of violent content (Mathiak and Weber, 2006^*^; Weber et al., 2006^*^). Lateral (especially left) prefrontal regions might be involved as well, integrating emotion and cognition and therefore working as a defense mechanism against negative emotions by down-regulating limbic activity (Montag et al., 2012). Wang et al. (2009) also provided evidence of that regulation mechanism by observing differing functional correlations between the left dlPFC and the ACC, and medial prefrontal regions & the amygdala during an executive task after a short-term exposure to a violent VG.

The reward circuit also seems to be implicated in the presence of violent content. Activation decreases in the OFC and caudate appeared in the absence of an expected reward. However, it does not seem that violence events were intrinsically rewarding (Mathiak et al. (2011^*^). Zvyagintsev et al. (2016^*^) found that resting-state functional connectivity was reduced within sensory-motor, reward, default mode and right frontotemporal networks after playing a violent VG, which could be linked to short-term effects on aggressiveness.

Gender differences in neural correlates were observed in one study (Chou et al., 2013^*^) after being exposed to violent content, with reduced blood flow in the dorsal ACC after playing a violent VG in males, but not females, possibly as a result of the role of the ACC in regulating aggressive behavior in males.

The effect of certain personality traits, particularly empathy, have been assessed using violent VG exposure (Lianekhammy and Werner-Wilson, 2015^*^). However, while empathy scores correlated with neural activity (frontal asymmetry during EEG), they were not affected by the presence of violent content. Markey and Markey (2010) found that some personality profiles, especially those with high neuroticism and low conscientiousness and agreeableness, are more prone to be affected by the exposure to violent VGs.

VG player's perspective may also be determinant to the level of moral engagement; while ERP N100 amplitudes were greater during a first person violent event, if the player was using a distant perspective, general alpha power was greater, which is indicative of lower arousal levels (Petras et al., 2015).

Montag et al. (2012), observed that regular gamers have been habituated to violence exposure and show less lateral prefrontal activation, linked to limbic down-regulation, compared to non-gamers. However, gamers have not lost the ability to distinguish real from virtual violence, as Regenbogen et al. (2010^*^) found, although that also depended on each person's learning history.

While attenuated P300 amplitudes have been linked to violence desensitization, both in short and long term exposure (Bartholow et al., 2006), these amplitudes did not increase using a pro-social VG (Liu Y. et al., 2015). Engelhardt et al. (2011), experimentally linked the lower P300 amplitudes to violence desensitization and their effects on aggression. Bailey et al. (2010) also supported the link between violent VG exposure and desensitization to violent stimuli, associating it with early processing differences in attentional orienting.

#### Flow

Flow and boredom states during VG play have also been the subject of research using neural correlates. The concept of flow, described by Csikszentmihalyi (1990), is understood as a mind state of being completely focused on a task that is intrinsically motivating. Among other characteristics, the state of flow implies a balance between the task difficulty and the person's skills, the absence of ambiguity in the goals of the task, and is commonly accompanied by a loss of awareness of time. Considering that the concept of flow is a complex construct which itself cannot be directly measured, it is necessary to operationalize its components. Some authors have identified some of these components as sustained attention (focus), direct feedback, balance between skill and difficulty, clear goals and control over the activity (Klasen et al., 2012^*^) and it has been theorized to be firmly linked to attentional and reward processes (Weber et al., 2009).

VGs provide the appropriate context in which flow states are encouraged to occur, since feedback is offered continuously and the level of difficulty is programmed to raise progressively, in order to match the improving skills of the player (Hunicke, 2005; Byrne, 2006). Therefore, VGs are perfect candidates to operationalize the components involved in the flow theory.

During gameplay in an action VG, Klasen et al. (2012^*^) could not relate the feedback component to any meaningful neural activity, but the four remaining flow-contributing factors showed joint activation of somatosensory networks. Furthermore, motor regions were implicated in the difficulty, sustained attention and control components. Together, the authors identify this sensorimotor activity as a reflection of the simulated physical activity present in the VG, which can contribute to the state of flow. The rest of the components elicited activity in several different regions. The reward system was involved in the skill-difficulty balance factor, observed by activation in the ventral striatum and other basal nuclei, rewarding the player in successful in-game events. In addition to activity in reward regions, this factor also correlated with simultaneous activity in a motor network comprised of the cerebellum and premotor areas. The factor comprising concentration and focusing during the VG was associated with changes in attentional networks and the visual system, as players switched away from spatial orientation to processing the numerous elements of the VG in high focus settings. Goal-oriented behavior showed decreased activity in the precuneus and regions of the ACC, while activity in bilateral intraparietal sulcus and right fusiform face area (associated with face processing) increased, which the authors explain as a result of a shift from navigation in a known environment to seeking new game content (Klasen et al., 2012^*^).

When manipulating the VG settings to elicit states or boredom, operationalized as the absence of goal-oriented behavior, one of the main aspects of flow, affective states appear. While the lack of goal-directed behavior resulted in an increase of positive affect, the neural correlates were characterized by lower activation in the amygdala and the insula (Mathiak et al., 2013). However, a different neural circuit was responsible when negative affect increased, characterized by activation in the ventromedial prefrontal cortex and deactivation of the HC and the precuneus, that seemed to counteract the state of boredom, possibly by planning future actions during inactive periods (Mathiak et al., 2013). Involvement of frontal regions was also observed by Yoshida et al. (2014) related to flow and boredom states. During the state of flow, activity in bilateral ventrolateral prefrontal cortex (vlPFC) [comprising the inferior frontal gyrus (IFG) and lateral OFC] increased, and it decreased when participants were subject to a boredom state. The OFC is linked to reward and emotion processing (Carrington and Bailey, 2009), and monitoring punishment (Kringelbach and Rolls, 2004). However, this study employed boredom differently, using a low difficulty level in the VG instead of the suppressing goal-directed behavior.

Brain-computer interfaces, using electrophysiological methods to measure brain activity, have been able to differentiate states of flow and boredom, created by adjusting the level of difficulty of a VG. The EEG frequencies that were able to discern between flow states were in the alpha, low-beta and mid-beta bands, measured in frontal (F7 and F8) and temporal (T5 and T6) locations (Berta et al., 2013).

### Gender differences

Although some studies have already discussed the presence of gender differences in cognitive processes related to VG playing, the lack of studies dealing with this topic and providing neural data are notable. The most relevant study of gender differences (Feng et al., 2007^*^) found that a 10-h training in an action VG (but not in a non-action VG) was enough to compensate for baseline gender differences in spatial attention, and to reduce the gap in mental rotation skills. Whether the initial difference was innate or a product of lesser exposure to this kind of activities in women is a matter of debate (Dye and Bavelier, 2010). Actually, one of the reasons men do not improve as much as women could be explained by a ceiling effect due to previous exposure to VGs. On the other hand, women with less experience in these activities are able to achieve equal performances in visuospatial skills that reach the same ceiling effect with a short training period. In this respect, Dye and Bavelier comment on the possible effects of lifetime VG exposure since the gender gap in attentional and non-attentional skills is smaller or non-existent during childhood compared to adult life, and the greater development of these skills in male individuals is partially due to games targeting a male audience.

Other authors (Ko et al., 2005) have focused on other psychosocial factors to explain gender differences in online VG addictions. Considering most online VGPs are men and this difference is also observed in addiction cases, they studied the possible factors and observed that lower self-esteem and lower daily life satisfaction are determinant in men, but not women. They attribute these differences to the reasons on why they play VGs: while men declared to play to pursue feelings of achievement and social-bonding, it was not the case for women. This aspect is not new to VG addiction and is shared aspect with other addictions. It is likely that VGs are used as a way to cope with these problems, leading up to the development of the addiction.

## Limitations

The study of neural correlates of VGs entails a number of inherent difficulties. The main limitation encountered during the development of this review was the dual nature of studies with regard to VGs as a research tool or as an object of study. The lack of standardization in study objectives is another limitation that should be addressed. Despite the recent popularity of VG-related studies, there are a multitude of similar research lines that offer hardly comparable results, making it difficult to draw general conclusions. We aimed to unify all sorts of studies in order to interpret and generalize the results.

First of all, we compared a large number of studies that not only used completely different techniques, but also had very heterogeneous research goals. We grouped them together with the aim of extracting all the available neuroimaging information, but it is likely that some information that would have been relevant for us was missed in the studies because their research objectives differed greatly from our own. In fact, in certain cases, VGs were almost irrelevant to the aim of the study and were only used as a substitute for a cognitive task, so the provided results may not directly reflect the VG neural correlates. Similarly, VGs were sometimes used as tools to provide violence exposure or to study the effects of behavioral addictions without the VG being the central object of study.

Another issue was the lack of a proper classification for VG genres. While the most common division is between action and non-action VGs, it would be interesting to establish which variables determine this classification. For instance, both first person shooters and fighting games could be considered action VGs. Both demand quick response times and high attentional resources, but first person shooter games require much higher visuospatial skills while fighting games do not. Consequently, efforts should be made to determine which aspects of each VG genre are related with each cognitive process and its associated neural correlates.

Apart from these aspects, comparisons between gamers and non-gamers are common in VG literature. Nevertheless, there is no consensus on the inclusion requirements for each group and it seems that no scientific criterion has been used to establish a cut-off line. Current dedication to VGs, measured in hours per week, seems to be the most common classification method. Non-gamer groups sometimes are so strict as to exclude any gaming experience, but on other occasions, for the same category, several weekly VG hours are tolerated. This is problematic since, in some cases, cognitive changes have been found after just a few weeks of VG training. However, in most cases, the onset age of active VG play, which is a particularly relevant aspect (Hartanto et al., 2016), is not taken into account. Another relevant variable, which tends to be forgotten, is lifetime VG experience, usually measured in hours. Moreover, despite the clearly different outcomes caused by different VG genres, this variable is not included when describing a participant's VG experience. Therefore, VG experience should be measured taking into account all the variables mentioned above: onset age, lifetime VG experience (in hours), current VG dedication (hours per week) and VG genres.

With regard to this review, it was really difficult to extract all the relevant information because of the limitations of the existing literature about the topic. But we did our best to clarify the results and to extract valuable conclusions.

Another limitation was the link between neural changes and cognitive functions. The neural correlates of VGs are the focus of this review, and we found it essential to complement this data by discussing their cognitive implications. In most cases these implications were directly assessed by the individual studies, but in some cases they were extrapolated based on previous literature. Furthermore, even when functional or structural changes are detected, they do not always reflect cognitive changes. This may be due to a lack of sensitivity in the cognitive and behavioral tasks employed. In order to detect both neural and cognitive changes, specific research designs, with sufficiently sensitive measurements of the three dimensions (functional, structural, and cognitive) are needed. Ideally, to determine when each change starts to appear as a result of VG exposure, an experimental design, including a VG training period, should be used. In this design, the neural and cognitive data would be assessed along a series of time points until the three types of changes were detected. An exhaustive discussion of the cognitive implications of VGs is beyond our scope since there are already other works that deal with this particular issue (Powers et al., 2013; Lampit et al., 2014; Toril et al., 2014; Wang et al., 2016).

Efforts should be made to systematize VG-related research, establishing VG training protocols and determining the effects of lifetime VG exposure, in order that more comparable results can be obtained and to improve the generalizability of results.

## Conclusions

The current work has allowed us to integrate the great deal of data that has been generated during recent years about a topic that has not stopped growing, making it easier to compare the results of multiple research groups. VG use has an effect in a variety of brain functions and, ultimately, in behavioral changes and in cognitive performance.

The attentional benefits resulting from the use of VG seem to be the most evidence-supported aspect, as many studies by Bavelier and Green have shown (Green and Bavelier, 2003, 2004, 2006, 2007, 2012; Dye et al., 2009; Hubert-Wallander et al., 2011; Bavelier et al., 2012b). Improvements in bottom-up and top-down attention, optimization of attentional resources, integration between attentional and sensorimotor areas, and improvements in selective and peripheral visual attention have been featured in a large number of studies.

Visuospatial skills are also an important topic of study in VG research, where optimization of cognitive costs in visuomotor task performance is commonly observed. Some regions show volumetric increases as a result of VG experience, particularly the HC and the entorhinal cortex, which are thought to be directly related to visuospatial and navigational skills. Optimization of these abilities, just like in attention and overall skill acquisition, is usually detected in functional neuroimaging studies as decreased activation in their associated pathways (in this case, in regions linked to the dorsal visual stream). It is likely that the exposure to a task first leads to an increase of activity in the associated regions, but ultimately, as the performance improves after repeated exposures, less cortical resources are needed for the same task.

Likewise, although not always consistent, even short VG training paradigms showed improvements in cognitive control related functions, particularly working memory, linked to changes in prefrontal areas like the dlPFC and the OFC. How to achieve far transfer in these functions remains one of the most interesting questions regarding cognitive control. Despite VGs being good candidates for cognitive training, it is still not well-known what the optimum training parameters for observing the first effects are. It seems intuitive that longer training periods will have a greater chance of inducing far transfer, but how long should they be? We also commented on how VG genre can have differential effects on cognitive control, so we cannot expect to observe these effects without first controlling this variable, since different VG genres often have little in common with each other.

Cognitive workload studies have offered the possibility of observing neural recruitment phenomena to compensate for the difficulty and complexity of a cognitive task and a number of studies have pointed to the importance of frontoparietal activity for this purpose.

It has been also possible to link skill acquisition rates with certain cerebral structures. Several brain regions are key in this regard, mainly the dlPFC, striatum, SMA, premotor area, and cerebellum. Moreover, as suggested by Anderson et al. (2015), models of whole-brain activation patterns can also be used as an efficient tool for predicting skill acquisition.

The role of the reward system is always present when we talk about VGs, due to the way they are designed. Addiction has a heavy impact throughout the neural reward system, including components like the OFC, the ACC, the ventral striatum, ventral pallidum, and midbrain dopaminergic neurons, together with diverse regions that have support roles in addiction. The role of structures that link addiction to its emotional components, such as the amygdala and the HC should not be underestimated. Limbic regions work together with the PCC to integrate the motivational information with the expectation of reward.

Exposure to violent content has implications regarding the reward circuits and also emotional and executive processing. Reduced functional connectivity within sensory-motor, reward, default mode and right frontotemporal networks are displayed after playing a violent VG. The limbic system, interacting with the lateral prefrontal cortex, has a role in down-regulating the reaction to negative emotions, like those found in violent contexts, which may lead to short-term violence desensitization.

Despite the difficulties in locating the main components of flow in the brain, it seems that several networks are involved in this experience. General activation of somatosensory networks is observed while being in this state, whereas activation in motor regions is only linked to three components of flow: skill-difficulty balance, sustained attention and control over the activity. The reward system has key implications in the experience of flow, showing that the ventral striatum and other basal ganglia are directly linked to the skill-difficulty balance in a task. When seeking new content in order to avoid boredom, the bilateral intraparietal sulcus and the right fusiform face area seem to be the most implicated regions. During a flow-evoking task, the absence of boredom is shown by activity in the IFC, the OFC, and the vmPFC. Flow is also linked to emotional responses, and both positive and negative affect during a VG have shown changes in the amygdala, insula, vmPFC and the HC.

It is also worth commenting on the negative effects of VGs. While much has been written about the possible benefits of VG playing, finding articles highlighting the negative outcomes in non-addicted or expert VGPs is much less common. To our knowledge, only four studies pointed out neural correlates which predicted hindered performance in a range of cognitive domains. VG use has been linked with reduced recruitment in the ACC, associated with proactive cognitive control and possibly related to reduced attentional skills (Bailey et al., 2010). Likewise, exposure to violent content in VG is associated with lower activity in the dlPFC, interfering with inhibitory control. The same team (Bailey and West, 2013) observed how VG play had beneficial effects on visuospatial cognition, but in turn had negative effects on social information processing. Lastly, VG exposition has been linked to delayed microstructure development in extensive brain regions and lower verbal IQ (Takeuchi et al., 2016).

Finally, although this review is focused on the neural correlates of VG, not their cognitive or behavioral effects, we believe in the importance of integrating all these aspects, since raw neuroimaging data often offer little information without linking it to its underlying cognitive processes. Despite the fact that this integration is increasingly common in the literature, this is not always the case and it is an aspect that could be addressed in future studies.

## Author contributions

All authors had an equal involvement during the process of making this review article. The article's design, data acquisition, and analysis of its content has been made by consensus among all the authors.

## Funding

This study has been supported by the doctoral school of the Open University of Catalonia, Spain, under the IN3-UOC Doctoral Theses Grants Programme 2013-2016 (http://in3.uoc.edu). The funders had no role in study design, data collection and analysis, decision to publish, or preparation of the manuscript.

### Conflict of interest statement

The authors declare that the research was conducted in the absence of any commercial or financial relationships that could be construed as a potential conflict of interest. The reviewer JMRA and handling Editor declared their shared affiliation, and the handling Editor states that the process nevertheless met the standards of a fair and objective review.
