# Supplementary material for: Neural Basis of Video Gaming: A Systematic Review
Source: Front Hum Neurosci. 2017 May 22;11:248. doi: 10.3389/fnhum.2017.00248 (PMC5438999; doi:10.3389/fnhum.2017.00248)
Supplement: Supplementary file 1 [file DataSheet1.docx]

# Appendix

## Keywords

### MEDLINE database

("Video game" OR "Videogame" OR "video-game" OR "video-gaming" OR "Video gaming" OR "Videogaming"

OR

"Casual gamer" OR "Casual gaming" OR "Hardcore gamer" OR "Hardcore gaming" OR "Online gamer" OR "Online gaming"

OR

"Serious gaming" OR "Serious game" OR "Serious games")

AND

("Neural Basis" OR "cortical" OR "cortex" OR "gray matter" OR "grey matter" OR "white matter" OR "matter volume" OR "Brain volume" OR "connectivity" OR "structural changes" OR "functional changes" OR "Activation patterns"

OR

"EEG" OR "ERP" OR "electroencephalography" OR "fMRI" OR "MRI" OR "Resonance" OR "TMS" OR "tDCS" OR "PET" OR "MEG" OR "NIRS" OR "fNIRS" OR "Near-infrared" OR "SPECT" OR "CT scan" OR "CAT scan" OR "tomography")

### Web of Science database

TS=("Video Game" OR “Videogame” OR "video-game" OR “video-gaming” OR “video gaming” OR “videogaming” OR “casual and gamer” OR “casual and gaming” OR “hardcore and gamer” OR “hardcore and gaming” OR “online and gamer” OR “online gaming” OR “serious gaming” OR “serious game” OR “serious games”) AND TS=("Neural basis" OR “cortical” OR “cortex” OR “gray matter” OR “grey matter” OR “white matter” OR “matter volume” OR “brain volume” OR “connectivity” OR “structural changes” OR “functional changes” OR “activation patterns” OR “EEG” OR “ERP” OR “electroencephalography” OR “fMRI” OR “MRI” OR “resonance” OR “TMS” OR “tDCS” OR “PET” OR “MEG” OR “NIRS” OR “fNIRS” OR “near-infrared” OR “SPECT” OR “CT scan” OR “CAT scan” OR “tomography”)

DOCUMENT TYPES: (ARTICLE OR ABSTRACT OR OTHER OR CLINICAL TRIAL OR LETTER OR CASE REPORT OR UNSPECIFIED)

## Glossary

ACC Anterior cingulate cortex

ACR Anterior corona radiata

BA Brodmann area

BG Basal ganglia

CBT Cognitive-Behavioral Therapy

CC Corpus callosum

CG Cingulate gyrus

CIAS Chen Internet Addiction Scale

CPEI Composite permutation entropy index

CST Corticospinal tract

dACC Dorsal anterior cingulate cortex

dlPFC Dorsolateral Prefrontal Cortex

DS Dorsal striatum

EC External capsule

EEG Electroencephalography

ERP Event-related potential

FA Fractional anisotrophy

FEF Frontal Eye Fields

FFA Fusiform face area

FG Fusiform gyrus

fMRI Functional magnetic resonance imaging

FPN Frontoparietal network

FPS First-person shooter

GM Gray matter

HC Hippocampus

IAD Internet Addiction Disorder

IAT Young’s Internet Addiction Test

IFG Inferior frontal gyrus

IFOF Inferior frontooccipital fasciculus

IGA Internet Gaming Addiction (subtype of IAD)

IGD Internet Gaming Disorder

ILF Inferior longitudinal fasciculus

IOG Inferior Occipital Gyrus

IPC Inferior parietal cortex

IPS Intraparietal sulcus

ITC Inferior temporal cortex

ITG Inferior temporal gyri

LIPFC Lateral inferior prefrontal cortex

lOFC Lateral orbitofrontal cortex

lOFG Lateral orbitofrontal gyrus

MCAs Middle cerebral arteries

MCC Middle cingulate cortex

MFC Middle frontal cortex

MFG Middle frontal gyrus

MMORPG Massively multiplayer online role-playing game

MOG Middle occipital gyrus

MRI Magnetic resonance imaging

MTC Middle temporal cortex

NIRS Near-infrared spectroscopy

OFC Orbito frontal cortex

OGA Online Gaming Addiction

PC Parietal cortex

PCA Posterior cerebral artery

PCC Posterior cingulate cortex

PCG Precentral gyrus

PET Positron emission tomography

PFC Prefrontal cortex

PHG Parahippocampal gyrus

POT Temporo-occipito-parietal

PPC Posterior parietal cortex

PTC Posterior temporal cortex

rACC Rostral anterior cingulate cortex

ReHo Regional homogeneity

RPG Role-playing game

SCR Superior corona radiata

SFG Superior Frontal Gyrus

SLF Superior longitudinal fasciculus

SMA Supplementary motor area

SN Salience network

SPC Superior parietal cortex

SPECT Single-photon emission computed tomography

SPG Superior parietal gyrus

SSVEP Steady state visually evoked potential

tDCS Transcranial direct current stimulation

TO Temporooccipital

TP Temporoparietal

VG Video game

VGP Video game Player

vmPFC Ventromedial prefrontal cortex

VRT Virtual Reality Therapy

VS Ventral striatum
